# Targeting DNA Damage Response and Immune Checkpoint for Anticancer Therapy

**DOI:** 10.3390/ijms23063238

**Published:** 2022-03-17

**Authors:** Jau-Ling Huang, Yu-Tzu Chang, Zhen-Yang Hong, Chang-Shen Lin

**Affiliations:** 1 Department of Bioscience Technology, College of Health Science, Chang Jung Christian University, Tainan 711, Taiwan; jaulingh@mail.cjcu.edu.tw (J.-L.H.); a0920215492@gmail.com (Y.-T.C.); zxc457659@gmail.com (Z.-Y.H.); 2 Graduate Institute of Medicine, College of Medicine, Kaohsiung Medical University, Kaohsiung 807, Taiwan; 3 Center for Cancer Research, Kaohsiung Medical University, Kaohsiung 807, Taiwan; 4 Department of Medical Research, Kaohsiung Medical University Hospital, Kaohsiung Medical University, Kaohsiung 807, Taiwan; 5 Department of Biological Sciences, National Sun Yat-sen University, Kaohsiung 804, Taiwan

**Keywords:** cancer immunotherapy, cGAS-STING, clinical trial, DNA damage response, immune checkpoint, PARP, synthetic lethality

## Abstract

Deficiency in DNA damage response (DDR) genes leads to impaired DNA repair functions that will induce genomic instability and facilitate cancer development. However, alterations of DDR genes can serve as biomarkers for the selection of suitable patients to receive specific therapeutics, such as immune checkpoint blockade (ICB) therapy. In addition, certain altered DDR genes can be ideal therapeutic targets through adapting the mechanism of synthetic lethality. Recent studies indicate that targeting DDR can improve cancer immunotherapy by modulating the immune response mediated by cGAS-STING-interferon signaling. Investigations of the interplay of DDR-targeting and ICB therapies provide more effective treatment options for cancer patients. This review introduces the mechanisms of DDR and discusses their crucial roles in cancer therapy based on the concepts of synthetic lethality and ICB. The contemporary clinical trials of DDR-targeting and ICB therapies in breast, colorectal, and pancreatic cancers are included.

## 1. Introduction

**Figure 1:**
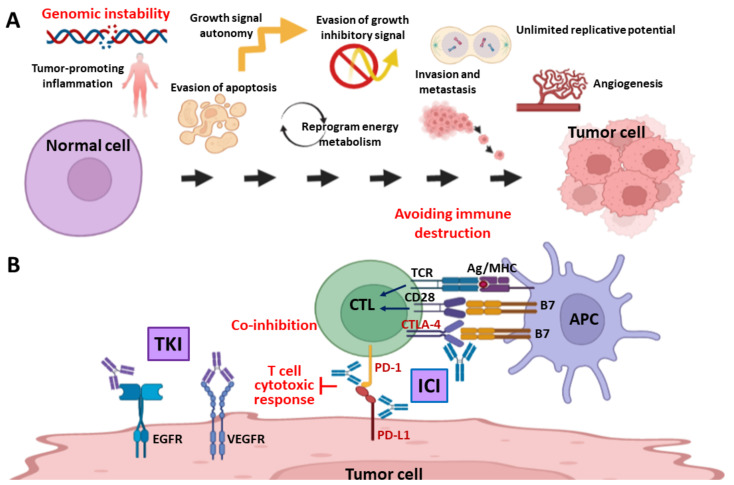
Cancer hallmarks, target therapy and immunotherapy. (**A**) Hallmarks and enabling characteristics of cancer. Hanahan and Weinberg propose six fundamental hallmarks (autonomous growth signal, evasion of growth inhibitory signal, evasion of apoptosis cell death, unlimited replicative potential, angiogenesis, and invasion and metastasis), two emerging hallmarks (reprogram energy metabolism and avoiding immune destruction), and two enabling characteristics (genomic instability and tumor-promoting inflammation) to distinguish cancer from normal cells. The genomic instability and avoiding immune destruction (in red) are the principal foundations of synthetic lethality and immune checkpoint blockade-based cancer therapy. (**B**) Target therapy and immunotherapy. (Left part) The epidermal growth factor receptor (EGFR) and vascular endothelial growth factor receptor (VEGFR) are the major targets of tyrosine kinase inhibitors (TKI), such as Herceptin^TM^ (trastuzumab) and Avastin^TM^ (Bevacizumab). (Right part) The CTLA-4, PD-1, and PD-L1 are immune checkpoint molecules (in red) for cancer immunotherapy. The immune checkpoint inhibitors (ICI) include ipilimumab (anti-CTLA-4 antibody), nivolumab and pembrolizumab (anti-PD-1 antibody), and atezolizumab, avelumab and durvalumab (anti-PD-L1 antibody). Ag, antigen. APC, antigen presenting cells. CTL, cytotoxic T lymphocyte. MHC, major histocompatibility complex. TCR, T-cell receptor. Graph created with Biorender.com.

Cancer is the major leading cause of death worldwide, accounting for nearly ten million deaths in 2020 [1]. Regardless of breakthroughs in anticancer therapies during the last few decades, a cure for cancer is still a challenge and the development of effective therapeutics is an urgent need for beating cancer. Considering the differences between cancerous and normal cells, Hanahan and Weinberg define important hallmarks of cancer, which include autonomous growth signal, evasion of growth inhibitory signal, evasion of apoptosis, unlimited replicative potential, angiogenesis, invasion and metastasis, reprograming energy metabolism and avoiding immunity-induced destruction (Figure 1A) [2,3]. Through deciphering these cancer hallmarks, scientists have developed target therapies by using small-molecule inhibitors and humanized monoclonal antibody (mAb) to battle with these cancer hallmarks, which provide alternative ways to treat cancer patients and may partly avoid side effects of chemotherapy. For examples, cell growth and angiogenesis signalings mediated by epidermal growth factor receptor (EGFR) and vascular endothelial growth factor receptor (VEGFR) pathways are identified as the main molecular targets of tyrosine kinase inhibitors (TKIs) (Figure 1B) [4,5]. Nowadays, dozens of target therapeutics have been approved to treat various cancers, including colorectal cancer (CRC), breast cancer (BC), melanoma, non-small cell lung cancer (NSCLC), leukemia, and so on [6,7]. However, not all patients are eligible to target therapy and drug resistance remains a problem to be overcome [8].

In addition to the aforementioned cancer hallmarks, Hanahan and Weinberg also point out two enabling characteristics: genomic instability and tumor-promoting inflammation, as crucial for cancer cells (Figure 1A). Dysregulation of DNA damage response (DDR) is an important driver of genomic instability [9,10]; in contrast, alterations of DDR genes can be biomarkers and therapeutic targets for cancer treatment [11,12,13]. Tumor-promoting inflammation reshapes the tumor immune microenvironment (TIME), which significantly influences the efficacy of cancer immunotherapy. A growing body of evidence demonstrates that targeting DDR is an attractive strategy to promote inflammation in TIME, which enhances immune recognition and the killing of malignant cells, especially when combined with an immune checkpoint inhibitor (ICI) (Figure 1B) [14,15]. However, because not all patients respond to such treatment, appropriate DDR and immune biomarkers are critical for patient selection. For example, deficiency in BRCA1 or BRCA2 gene is observed to be associated with improved response to ICIs in patients with biliary tract cancer or hepatocellular carcinoma [16,17]. This review introduces the DDR and DNA repair mechanisms, the strategy of targeting DDR to enhance ICI efficacy, and contemporary clinical trials of DDR inhibitors and ICIs, either used as monotherapy or combination therapy, in breast, colorectal, and pancreatic cancers.

## 2. DNA Damage Response (DDR) and DNA Repair Systems

Upon DNA damage, cells can detect DNA lesions through several sensors and kinases, such as ataxia telangiectasia mutated (ATM), ataxia telangiectasia and Rad3-related (ATR), and DNA-dependent protein kinase (DNA-PK), to launch DDR [18]. These serine/threonine kinases elicit DDR through protein phosphorylation cascades and, as results, cells may undergo DNA repair, cell cycle arrest, gene expression, or apoptosis, depending on the severity of DNA damage and efficiency of DNA repair [19,20,21]. In this review, we focus on DDR-mediated cell cycle arrest and DNA repair, in which several genes can serve as biomarkers and therapeutic targets in terms of cancer immunotherapy [13,14,22,23,24,25].

**Figure 2:**
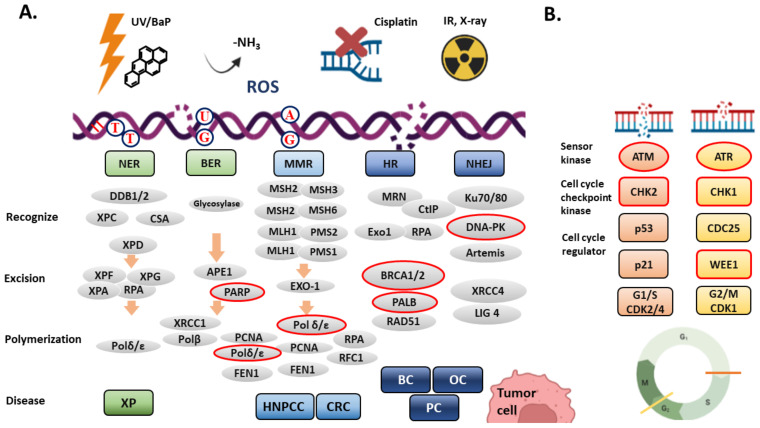
(**A**) DNA repair systems. Different DNA damages are recognized and repaired through different DNA repair pathways. DNA damages shown here includes ultraviolent light (UV)-induced cyclobutane pyrimidine dimers (TT), benzo[a]pyrene (BaP)-induced bulky DNA adducts, DNA interstrand crosslink, deamination (-NH3, GC to GU pairing), reactive oxygen species (ROS) induced 8-oxoguanine, mismatch DNA pairing (e.g., AG pairing), double-strand breaks (DSBs) induced by ionized radiation, X-ray or cisplatin. Except for direct repair, nucleotide excision repair (NER), base excision repair (BER), mismatch repair (MMR), homologous recombination (HR) and non-homologous end-joining (NHEJ) are shown here. Each DNA repair mechanism comprises three main procedures: recognition, excision, and polymerization. The red circle indicates the defective mutations found in hereditary nonpolyposis colorectal cancer (HNPCC), colorectal cancer (CRC), breast cancer (BC), ovary cancer (OC) and pancreatic cancer (PC). (**B**) The DNA damage response (DDR). ATM and ATR are activated by DSBs and single-strand breaks. The activated ATM and ATR phosphorylate downstream cell cycle checkpoint kinases, CHK1 and CHK2, which then phosphorylate p53, CDC25, and WEE1. The phosphorylated p53 increases the expression of p21, a potent CDK inhibitor. Phosphorylation of CDC25 (inactive) and WEE1 (active) results in inhibition of CDK activity and leads to cell cycle arrest at G1/S and G2/M transition. Graph created with Biorender.com.

Cells have invested several DNA repair systems to deal with different kinds of DNA damages [26] (Figure 2A). The simplest way of DNA repair is direct repair, which uses the alkyltransferase and O^6^-methylguanine-DNA methyltransferase (MGMT) to directly remove alkylations from DNA [27]. The expression and promoter methylation of MGMT in glioblastoma can serve as a predictive biomarker for the response to temozolomide (an alkylating agent) treatment. Low expression or promoter hypermethylation of MGMT is correlated with a favorable outcome of glioblastoma patients who received temozolomide, because these cancer cells are unable to efficiently repair temozolomide-induced alkylations [28,29].

In 2015, the Nobel Prize in chemistry was awarded to Tomas Lindahl, Paul Modrich and Aziz Sancar “for mechanistic studies of base excision repair (BER), mismatch repair (MMR) and nucleotide excision repair (NER)”, respectively [30]. The basic mechanisms, biomarker roles, and therapeutic implications of these DNA repair pathways are briefly summarized below:

### 2.1. Nucleotide Excise Repair (NER)

NER is involved in the repair of ultraviolet light (UV)-induced cyclobutane pyrimidine dimers (CPD) and pyrimidine (6–4) pyrimidone photoproducts (6–4 PP), as well as bulky DNA adducts induced by chemotherapy drugs (e.g., cisplatin) and genotoxic pollutants (e.g., polycyclic aromatic hydrocarbons). The repair process comprises three steps (recognition, excision, and polymerization) to fix the DNA lesions [27,31]. Global genome (GG) and transcription-coupled (TC) NER sub-pathways are different at recognition step and use different recognition proteins, DDB-XPC-hHR23B and RNA polymerase II-CSA, respectively. Next, the excision and polymerization steps recruit single-strand binding protein RPA, helicase XPD, endonucleases XPF and XPG. XPF-associated excision repair cross-complementing protein 1 (ERCC1) and ERCC4, and polymerases δ/ε to excise DNA lesions and synthesize new DNA strand. Finally, the DNA nick between newly synthesized and original DNA ends is joined together by DNA ligase 3 to complete the repair. Germline mutations of NER genes, such as Xeroderma pigmentosum (XP) family genes, are associated with increased cancer incidence in the affected patients [9,32]. In NSCLC, low expression of ERCC1 predicts a better outcome in patients who receive platinum-based chemotherapy due to inefficient NER and increased apoptosis of tumor cells [33,34].

### 2.2. Base Excision Repair (BER)

BER acts on the oxidative DNA bases (e.g., 8-oxyguanine) and apurinic/apyrimidinic (AP) sites caused by endogenous deamination (C to T) or depurination reactions. BER divides into short- and long-path sub-pathways [27,31]. Short-patch BER is mediated by DNA glycosylase and AP endonuclease APE1. Then, the poly(ADP-ribose) polymerase 1 (PARP1) binds to BER intermediates and transfers poly(ADP-ribose) to DNA, BER effectors, and histones (PARylation) for recruiting XRCC1 and polymerase β to repair the aberrant nucleotides [35,36]. The long-patch BER involves proliferating cell nuclear antigen (PCNA), FEN1 endonuclease, and DNA polymerase δ/ε to remove and displace DNA lesions (2–10 bases). Finally, the DNA ligases 1 (long-patch) and 3 (short-patch) seal the gaps on the single-strand breaks (SSBs).

The PARylation activity of PARP1 is involved not only in BER but also in other DNA repair mechanisms. Auto-PARylation of PARP1 keeps itself away from DNA due to the negative charge of poly(ADP-ribose) chains. Inhibition of PARylation results in the “trapping” of PARP1 on DNA, which will induce the collapse of DNA replication forks when the DNA replication machinery encounters the trapped PARP–SSB complexes [36,37,38,39]. Thus, inhibition of PARP1 impairs the BER process that leads to the accumulation of SSBs and, subsequently, induces DSBs during DNA replication, which requires additional mechanisms to repair (Section 2.4).

### 2.3. Mismatch Repair (MMR)

MMR deals with mismatch nucleotides of replication errors [27,31]. The recognition of mismatch nucleotides is performed by the heterodimeric protein complexes MSH2/3 and MSH2/6. Then, the MLH1/PMS2 and MLH1/PMS1 are recruited to the mismatch sites to facilitate the removal of mismatch nucleotides and the synthesis of a new DNA strand by exonuclease 1 (EXO1), DNA polymerases δ/ε, RPA, PCNA, RFC, and FEN1. Hereditary non-polyposis colorectal cancer (HNPCC) is one of the most common cancer syndromes in humans. Half of HNPCC patients carry germline mutations in MLH1 or MSH2. Deficiency of MMR (dMMR) is correlated with microsatellite instability (MSI). High levels of MSI (MSI-H) have been found in many different types of cancer [40,41]. Both MSI-H and dMMR are important biomarkers for personized cancer immunotherapy [42].

### 2.4. Duble-Strand Break (DSB) Repair and the Cell Cycle Checkpoint

Severe DSBs are fatal to proliferative cells and, thus, need to be efficiently repaired. Non-homologous end-join (NHEJ) and homologous recombination (HR) repair are the most important mechanisms to repair DSBs, which can be induced by replication stress, ionized radiation, and anticancer agents, such as cisplatin, mitomycin C, camptothecin, and DNA replication inhibitors [27,31].

Upon DSBs, ATM and DNA-PK are activated immediately to initiate DDR through HR and NHEJ pathways, respectively. ATR is activated by replication stress-induced SSBs, which are coated by RPA and ATRIP [27,31]. Activation of these kinases can phosphorylate many DSB repair proteins, such as NBS1 and BRCA1, and cell cycle regulators including the cell cycle checkpoint kinase 1 and 2 (CHK1 and CHK2) and the tumor suppressor p53. CHK1 and CHK2 also phosphorylate p53, which then upregulates the expression of the CDK inhibitor p21 and pauses the cell cycle progression. In addition, CHK1 and CHK2 phosphorylate CDC25, which leads to an export of phosphorylated CDC25 from the nucleus to the cytoplasm. Because CDC25 removes the inhibitory phosphate groups from CDKs in the nucleus, CHK1- and CHK2-mediated cytoplasmic transportation of CDC25 results in CDK inactivation and cell cycle arrest. In contrast to the role of CDC25 in CDK1 activation, the WEE1 kinase phosphorylates and inactivates CDK1; therefore, the cell cycle progression from G2 to M phase is inhibited [43] (Figure 2B). Inhibition of WEE1 may result in mitosis entry with DNA lesions or under-replicated DNA, which subsequently causes mitotic catastrophe and cell apoptosis. The first small molecule inhibitor of WEE1 kinase (Adavosertib) is under clinical trials in uterine carcinoma, acute myeloid leukemia, and RAS/TP53-mutated metastatic CRC [44].

DSB repair through HR requires sister chromatids as templates; therefore, HR repair (HRR) only takes place after the cells undergo DNA replication in S phase and the onward G2/M phases. In contrast, NHEJ does not require template DNA for repair and serves as the major DSB repair mechanism in G1 phase [45]. When DSBs appear in G1 phases, the heterodimer Ku70/80 protein complex binds to the lesions immediately and works together with DNA-PK to recruit the endonuclease Artemis for end processing of DSBs. Next, the processed broken ends are joined together by the DNA ligase 4/XRCC4 complex [45]. In HRR, the ATM kinase phosphorylates itself, histone H2A.X, and NBS1, which is one component of the MRE11-RAD50-NBS1 (MRN) complex for recognition of DSBs. The MRN complex, C-terminal binding protein interacting protein (CtIP), exonuclease EXO1, and BRCA1 are involved in DNA resection and 53BP1 de-phosphorylation [46]. The resected single-strand DNA protrusion is protected by RPA and then can invade into the complementary sister chromatid DNA by the assistance of RAD51, BRCA2, and Partner And Localizer Of BRCA2 (PALB2) for HR [47].

In HRR pathway, germline or somatic BRCA1 and BRCA2 mutations are associated with increased risks of solid tumors, especially breast and ovary cancers [48]. Loss-of-function mutations of ATM, CHK1, CHK2, RAD51C and RAD51D can also be found in familial breast and ovary cancers as well as in pancreatic cancer [49,50,51]. PALB2 has been reported as a susceptibility gene for pancreatic cancer [52,53,54,55].

## 3. Targeting DDR—Induction of Synthetic Lethality in Cancer Cells

DDR is a double-edged sword, which keeps genome stability in normal cells and prevents the birth of cancerous cells; on the other hand, it helps cancer cells resist therapy-induced DNA damage and cell death. Therefore, inhibition of DDR in cancer cells can theoretically enhance the efficacies of numerous anticancer therapeutics. In this regard, synthetic lethality is proven a promising strategy to inhibit cancer cells that harbor deficient BRCA1 or BRCA2 genes [56,57,58].

**Figure 3:**
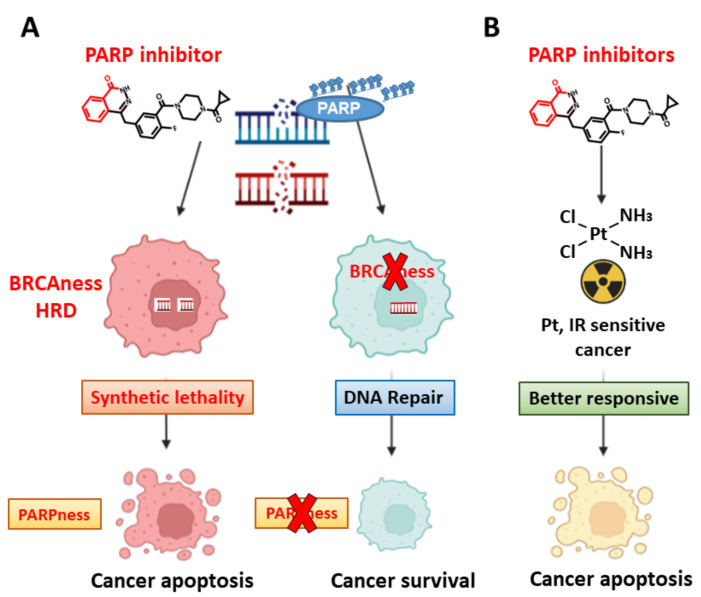
(**A**) Synthetic lethality by BRCAness and PARPness. The core structure (phthalazinones) of PARP inhibitor olaparib is shown in red. Inhibition of PARP in cancer cells harboring BRCA1/2 mutation or homologous recombination repair deficient (HRD) phenotype leads to DNA double-strand breaks (DSBs) and cell apoptosis. This condition is PARP sensitive (PARPness) (left part). Auto-PARylation of PARP is essential for base excision repair and DSB repair to survive (central part). (**B**) Synthetic lethality beyond BRCAness and PARPness. PARP inhibitor increases cell apoptosis in cisplatin or radiotherapy sensitive cancers, suggesting that PARP inhibitor can be a sensitizer of cisplatin and ionizing radiation. Graph created with Biorender.com.

As mentioned previously in Section 2.2, targeting PARP1 will increase SSBs and subsequently DSBs after DNA replication. These DSBs can be repaired in normal cells with proficient HRR; however, DSBs cannot be efficiently repaired in BRCA1- or BRCA2-mutated breast and ovarian cancer cells due to HRR deficiency (HRD). Thus, BRCA1- or BRCA2-mutated cancer cells exhibit an increased sensitivity to PARP inhibitors when compared to normal cells [38,56,57] (Figure 3A). In addition to BRCA1 and BRCA2, mutations of other HRR genes that cause HRD may also confer a phenotype (BRCAness) similar as that of BRCA mutations [59]. Therefore, cancer cells that harbor HRR gene mutations and show HRD or BRCAness phenotype will be sensitive to PARP inhibitors (PARPness) [60,61,62,63].

Olaparib is the first FDA approved PARP small molecule inhibitor for BRCA1- and BRCA2-mutated cancers [64,65]. At present, olaparib is approved for germline BRCA-mutated (gBRCAm) advanced ovary, breast, and pancreatic cancers and HRR gene-mutated metastatic castration-resistant prostate cancer (mCRPC). In addition to olaparib, many PARP inhibitors are under clinical trials and rucaparib, niraparib, and talazoparib have been approved by FDA and EMA for cancer therapy. Talazoparid exhibits the most potent PARP1 trapping effect. Although these PARP inhibitors show good efficacy and specificity in gBRCAm cancers, they should be used carefully to avoid severe adverse effects [66].

In HR-proficient cancers, induction of synthetic lethality can be achieved through HRR inhibition by using specific inhibitors of ATM, ATR, Topoisomerase 2 or Bromodomain Containing 4 (BRD4) to create a BRCAness phenotype. BRD4 recognizes acetyl-lysine residues on histone proteins and plays a crucial role in regulating chromatin conformation to keep genome stability. CtIP is an endonuclease that cooperates with MRN complex to recruit BRCA 1 on DSB sites (Figure 2A). Regardless of mutation status of BRCA1/2, inhibition of BRD4 leads to depletion of CtIP and causes HRD. Therefore, combination of PARP and BRD4 inhibitors can induce synthetic lethality in HR-proficient cancers [67,68]. Similarly, inhibition of other HRR genes, such as ATM and ATR, leads to BRCAness and sensitizes cancer cells to PARP inhibitors [69]. There are several inhibitors of ATM, ATR, CHK1, DNA-PK, WEE1 are under clinical development for treating solid tumors and lymphoma [29,44,70]. Nowadays, induction of synthetic lethality in cancer cells has become a promising strategy in cancer therapy [13,71].

PARP inhibitors can also be combined with traditional anticancer therapeutics [72] (Figure 3B). The sensitivity of PARP inhibitor are observed in cancer patients who are sensitive to platinum drugs or radiotherapy [73]. PARP inhibitors in combination with platinum chemotherapy elicit a higher response rate in advanced pancreatic cancer patients than chemotherapy alone [74]. This combination synergistically induces apoptosis in cancer cells.

## 4. Targeting DDR—Stimulation of Immune Response through Generation of Cytosolic DNA

**Figure 4:**
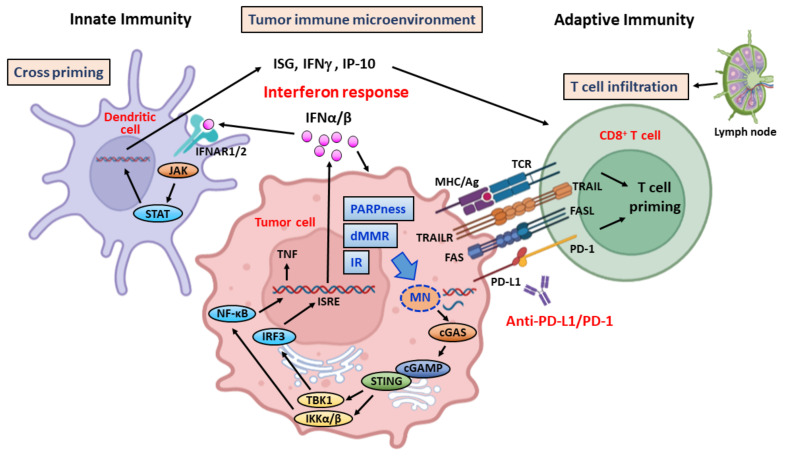
Cytosolic DNA sensing by cGAS-STING is crucial for immunotherapy. Ionizing radiation (IR), mismatch repair deficiency (dMMR), and PARP inhibitor-induced synthetic lethality (PARPness) in cancer cells induce DNA damage that increase micronuclei (MN) and amounts of cytosolic DNA. The cyclic GMP-AMP synthase (cGAS) binds to cytosolic DNA and produces cyclic-dinucleotide, 2′3′-cGAMP (cGAMP), which activates the stimulator of interferon genes (STING), allowing STING translocation from endoplasmic reticulum to Golgi apparatus to activate interferon regulatory factor 3 (IRF3) and NF-κB through the TANK-binding kinase 1 (TBK1) and IKKα/β. The activated IRF3 and NF-κB go into nucleus to turn on expression of interferon α/β (IFNα/β) and TNF-α through interferon–stimulated responsive element (ISRE) and NF-κB responsive element. IFNα/β bind to interferon receptor (IFNR1/2) to activate JAK/STAT signaling and produce interferon-stimulated genes (ISGs), IFNγ, and IP-10, which can activate and attract CD8+ T cells migration from lymph nodes to tumor microenvironment to kill cancer cells via FAS/FASL and TRAIL/TRAILR interactions. Graph created with Biorender.com.

PARP and other DDR inhibitors can elicit DSBs and genomic instability, which increase the formation of micronucleus (MN) and cytosolic DNA in cancer cells. The DSB- and MN-derived cytosolic DNA is detected by pattern recognition receptors (PRRs), which are members of innate immunity to sense pathogenic nucleic acids, such as virus DNA or RNA. Similar as the case of virus infection, cytosolic DNA detected by PRRs will activate interferon (IFN) signaling and subsequent innate and adaptive immune responses (Figure 4).

The cyclic GMP-AMP synthase (cGAS) is one of the major PRRs to detect cytosolic DNA in cancer cells [75,76]. Upon binding of cytosolic DNA, cGAS catalyzes the synthesis of cyclic-dinucleotide 2′3′-cGAMP (cGAMP), which binds to the stimulator of interferon genes (STING) and leads to STING translocation from endoplasmic reticulum to Golgi apparatus. The binding of cGAMP to STING activates downstream signaling molecules include TANK-binding kinase 1 (TBK1)/interferon regulatory factor 3 (IRF3) and IKKα/β/NF-κB to produce type I IFN (IFN-I), IFN-stimulated genes (ISGs), and pro-inflammatory cytokines, such as TNF-α [77,78]. The secreted IFNs bind to the heterodimer type I IFN receptors (IFNAR1/IFNAR2) on the dendritic cells (DCs) and activate JAK/STAT signal pathway to express ISGs and pro-inflammatory cytokines, including IFNγ and IP-10 (CXCL10), which contribute to the activation and tumor infiltration of CD8+ T cells. In addition to DCs, IFNs also regulate a lot of innate and adaptive immune cells, such as natural killer (NK) cells, macrophages, plasma B cells, CD8+ cytotoxic and CD4+ helper T cells, and modulate their maturation, migration, and activation [79]. These IFN-induced immune responses are crucial for the killing of cancer cells; thus, IFN response induced by DDR targeting (PARPness) can enhance the efficacy of cancer immunotherapy [80,81]. Furthermore, in dMMR or ionizing irradiated tumor cells, which produce more cytosolic DNA, cGAS-STING-IFN pathway has been demonstrated to play an important role in triggering antitumor activity (Figure 4) [75,82,83,84]. In mouse models, using tumor cells-produced IFNs can increase specific immunity against primary tumors, enhance DC cross-priming and increase infiltration and effector function of CD8+ T cells in the tumor microenvironment [85]. In contrast, loss of STING-IFNs signaling in cancer cells constrains antitumor T-cell priming. These results show that impaired cGAS-STING-IFN responses may enable cancer cells to evade immune surveillance [86,87].

In cancer immunotherapy, the cytotoxic T lymphocytes (CTLs) or CD8+ T cells play a critical role in the killing of cancer cells after stimulation by tumor antigens (Figure 4) [88]. DCs are professional antigen presenting cells (APCs) that present cancer-associated antigens, including genetic alterations-derived neoantigens, together with major histocompatibility complex (MHC) to CD8+ T cells. In addition to MHC molecules and peptide antigens, co-stimulatory signals are required for activation of CD8+ T cells. The principal co-stimulatory molecules on APCs are B7 molecule, which interacts with CD28 molecule on the T-cell surface. This signal induces clonal expansion and activation of CTLs only when the co-stimulatory signal is also given by the binding of CD28 to B7 (Figure 1B).

The activity of CTLs is tightly regulated by an immune checkpoint mechanism to avoid severe inflammation and tissue damage, which are observed in autoimmune diseases. Cytotoxic T-lymphocytes-associated protein 4 (CTLA4) is a B7-inhibitory and complementary receptor on activated T cells. It binds to B7 with a 20-fold higher affinity than that of CD28. Interaction of B7 and CTLA4 acts as a brake that inhibits the activation and proliferation of T cells [89,90]. PD-1 is another inhibitory receptor on the cell surface of T cells. The PD-1 ligands, PD-L1 and PD-L2, can be expressed by malignant cells after exposure to pro-inflammatory cytokines (Figure 1B). High-expression of CTLA-1, PD-L1, and PD-L2 immune checkpoint molecules in cancer cells is an important mechanism underlying immune escape of cancer cells [88].

In 2011, FDA has approved the anti-CTLA-4 mAb (ipilimumab) for treating melanomas [91]. Together with anti-PD-1 (nivolumab and pembrolizumab), anti-PD-L1 (atezolizumab, avelumab, and durvalumab) mAbs, the use of these ICIs opens a new era in cancer therapy. Since then, many clinical trials are set to evaluate the efficacies of ICIs, either used as monotherapy or combination therapy, on various cancer types. For metastatic melanoma as an example, patient’s long-term survival rate can be extended from 5% to 50% with anti-PD-1 plus anti-CTLA-4 combination therapy [92]. Nevertheless, only a minority of cancer patients are responsive to immune checkpoint blockade (ICB) therapy because cancer cells may evolve several mechanisms to escape ICI-based therapy. These immune escape mechanisms are related to the lack of neoantigens, impaired intra-tumor immune infiltration, impaired IFNγ signaling, and severe T-cell exhaustion, which exhibit a “cold” TIME [93,94]. To overcome this situation, combinations with other therapeutics that can reshape TIME from “cold” to “hot” may improve the efficacy of immunotherapy. The candidate therapeutics for inducing “hot” TIME include chemotherapy, radiotherapy, epigenetic modifiers, immune stimulation agent, and target therapy. Among them, DDR-targeting are expected to show high potency in combination with ICIs, and some clinical trials exploiting this combination are underway (see next Section 5).

Radiotherapy not only generates cytotoxic DNA damage but also induces tumor-specific immune response, which enhances anticancer efficacy [95]. It has been shown that ionized radiation can activate type I IFN response and recruit CD8+ cytotoxic T cells to TIME [96]. These effects are related to the generation of cytosolic DNA and the activity of cGAS-STING-IFN pathway. In a cGAS-knockout mouse model, PD-L1 blockade lost its antitumor effects. It has been shown that cGAMP exhibits a strong antitumor effect, especially when it is combined with the PD-L1 antibody [76]. Several STING agonists, such as ADU-S100 and MK-1454 are tested as sensitizers for immunotherapy [97,98,99]. Wang et al. have shown that the treatment of PARP inhibitor pamiparib increases PD-L1 expression in pancreatic cancer cells and the combination of pamiparib and PD-L1 mAb improved therapeutic efficacy when compared to monotherapy [100]. These results support the notion that combination of ICIs and DDR-targeting therapeutics can improve the efficacy of cancer immunotherapy.

The tumor-intrinsic DDR status, such as tumor mutational burden (TMB), MSI, dMMR, or HRD, can also influence the efficacy of ICI-based immunotherapy [22,25,82]. Therefore, tumor-intrinsic DDR features can serve as biomarkers to select suitable patients for immunotherapy. In 2017, FDA first approved the use of pembrolizumab in the patients with dMMR or MSI-H in their cancers regardless of histological cancer types [101,102]. The cGAS-STING signaling can contribute to ICI-based therapy in DDR-altered tumors [76,82,103]. As previous mentioned, IP-10 induced by cGAS-STING-IFN pathway plays an important role in chemotaxis of cytotoxic T cells to TIME [104]. In a study containing 1310 breast cancer patients, IP-10 is upregulated in HRD tumors and is associated with high neoantigen load and increased infiltrating immune cells. Moreover, the expression of IP-10 can serve as a biomarker for the response to anti-PD-1/PD-L1 therapy [105]. Therefore, elucidating the relationship between tumor-intrinsic DDR characteristics and ICI efficacies can improve the development of precision cancer immunotherapy [23,80,106].

## 5. Targeting DDR and Immune Checkpoint—Contemporary Clinical Trials in Breast, Colorectal, and Pancreatic Cancers

As described above, only a subgroup of patients with specific genetic contexts (dMMR/MSI-H) have a better response rate to ICB therapy [101,102]. Thus, DDR alterations are useful biomarkers for choosing the patients to receive ICB therapy [106,107]. Alternatively, targeting DDR mechanisms through the use of PARP and other DDR inhibitors is also a promising way to induce a “hot” TIME to improve the efficacy of cancer immunotherapy. At present, dozens of clinical trials are ongoing to assess the improvement effects of DDR-targeting therapeutics on ICB therapy. This review summarizes the current states of these clinical trials in breast, colorectal, and pancreatic cancers.

### 5.1. Breast Cancer

Breast cancer was the most common cancer in 2020 (2.26 million cases). It is estimated that around 5–10% of breast cancers are inherited with gene mutations, which include germline mutations of BRCA1, BRCA2, and other DDR genes [55,108]. Therefore, breast cancer can be a paradigm of DDR-targeting and ICB therapies (Table 1).

#### 5.1.1. Combination Therapy

##### MEDIOLA Trial (NCT02734004)

This is a multicenter, open label, phase 1/2 basket trial to evaluate the efficacy and safety of olaparib (PARPi) in combination with durvalumab (anti-PD-L1 mAb) in 264 patients with advanced solid tumors, which include metastatic and HER2-negative breast cancer containing germline BRCA mutation (gBRCAm), relapsed ovarian cancer with gBRCAm, relapsed small cell lung cancer (SCLC), and gastric cancer (initial stage cohorts). In the patients with gBRCAm metastatic breast cancer, the combination of olaparib and durvalumab shows promising antitumor activity. The side effects of this combination regimen are similar to that of olaparib or durvalumab monotherapy. The long-term therapeutic benefit of this combination therapy in patients with gBRCAm is waiting for the next phase clinical trial [109].

##### TOPACIO Trial (NCT02657889, KEYNOTE-162)

This multicenter, open label, phase 1/2 trial evaluated the efficacy and safety of niraparib (PARPi) in combination with pembrolizumab (anti-PD-1 mAb) in 122 patients with advanced or metastatic triple-negative breast cancer (TNBC) or recurrent ovarian cancer. In the 55 TNBC patients, the trial reveals that niraparib plus pembrolizumab provides promising antitumor activity, especially for those with BRCA mutations. The combination therapy has a tolerable safety profile [110,111].

##### TALAVE Trial (NCT03964532)

This is a multicenter, open label, phase 1/2 pilot trial of induction talazoparib (PARPi) followed by the combination of talazoparib and avelumab (anti-PD-L1 mAb) in advanced breast cancer. Avelumab is known to activate NK cells and modulate both adaptive and innate immune mechanisms [112]. The trial enrolled 24 participates. The primary and secondary outcomes are treatment-related adverse events and overall response rate (ORR), respectively. Other outcome measurements include progression free survival (PFS), overall survival (OS), duration of response (DOR), and disease control rate (DCR). PD-L1 expression will be evaluated by immunohistochemistry (IHC).

##### NCT02849496 

This trial selected BRCA mutant, non-HER2-positive breast cancer patients. The trial tested the efficacies of olaparib (PARPi) and atezolizumab (anti-PD-L1 mAb), either in the setting of monotherapy or combination therapy.

**Table 1:** Clinical trials of breast cancer using immunotherapy or synthetic lethality strategy.

Treatment	DDRi	Target	ICI	Target	Disease Setting	Biomarker	Trial ID	Phase	Comment/Reference
Combination	Olaparib	PARP	Durvalumab	PD-L1	Metastatic HER2- BC ^1^	Germline BRCAm ^2^	NCT02734004	II	MEDIOLA trial [109]
	Niraparib	PARP	Pembrolizumab	PD-1	aTNBC ^3^ or mTNBC ^4^	BRCAm	NCT02657889	II	TOPACIO trial [111]
	Talazoparib	PARP	Avelumab	PD-L1	aBC ^5^		NCT03964532	I/II	TALAVE trial [112]
	Olaparib	PARP	Atezolizurmab	PD-L1	aHER2- BC or mHER2- BC	BRCAm, HRD ^6^	NCT02849496	II	
	Olaparib	PARP	-	-					
	Olaparib	PARP	Pembrolizumab	PD-1	aTNBC or mTNBC	CT+ICI positive ^7^	NCT04191135	II/III	KEYLYNK-009 trial [113]
	v.s. CT (C+G) ^8^	DNA	Pembrolizumab	PD-1					
Monotherapy DDRi or ICI	CT (nP) ^9^		Atezolizurmab	PD-L1	Untreated mTNBC		NCT02425891	III	IMpassion 130 trial [114]
	CT (nP)		v.s. Placebo						
			Durvalumab	PD-L1	NACT Resist TNBC ^10^		NCT03740893	II	Phoenix trial
	AZD6738	ATR							
	Olaparib	PARP							
Monotherapy DDRi or ICI (molecular selection)	Talazoparib	PARP			aBC and/or mBC ^11^	Germline BRCAm	NCT01945775	Approved	EMBRACA trial [115,116]
	Olaparib	PARP			mBC	Germline BRCAm	NCT02000622	Approved	OlympiAD trial [117]
			Pembrolizumab	PD-1	mBC, etc.	BRCAm, POLD1m ^12^, POLEm ^13^	NCT03428802	II	

^1^ BC = Breast Cancer; ^2^ BRCAm = BRCA1 or 2-mutated; ^3^ aTNBC = Advanced triple-negative breast cancer; ^4^ mTNBC = Metastatic Triple-Negative Breast Cancer; ^5^ aBC = Advanced Breast Cancer; ^6^ HRD = Homologous Recombination Deficient; ^7^ CT + ICI positive = Clinical benefit with first-line CT (Carboplatin and Gemcitabine) Plus Ici; ^8^ CT (C + G) = Carboplatin and Gemcitabine chemotherapy; ^9^ CT (nP) = Nanoparticle albumin-bound paclitaxel chemotherapy;^10^ NACT Resist TNBC = Neoadjuvant Chemotherapy Resistant Residual Triple Negative Breast; ^11^ mBC = Metastatic Breast Cancer; ^12^ POLD1m = POLD1 Mutated; ^13^ POLEm = POLE Mutated.

##### KEYLYNK-009 Trial (NCT04191135)

This is an open-label, randomized, phase 2/3 study of olaparib (PARPi) plus pembrolizumab (anti-PD-1 mAb) versus chemotherapy plus pembrolizumab. The 1225 participants with metastatic triple-negative breast cancer (TNBC) have previous induction chemotherapy plus pembrolizumab. The chemotherapy drugs (carboplatin and gemcitabine) and olaparib can induce DNA damage and may also activate cGAG-STING signaling.

#### 5.1.2. Monotherapy

##### IMpassion130 Trial (NCT02425891)

This is a randomized, placebo-controlled, double-blind, phase 3 trial including 902 previously untreated metastatic TNBC patients in 41 countries to evaluate atezolizumab (anti PD-L1 mAb) plus nab-paclitaxel versus nab-paclitaxel. The trial revealed a clinically meaningful overall survival (OS) benefit with atezolizumab plus nab-paclitaxel in patients with PD-L1 immune cell-positive disease. However, no significant difference in OS was observed between treatment groups due to no biomarker-guided patient stratification. This indicates that before immunotherapy, the personalize gene analyzed is important [118,119].

##### PHOENIX Trial (NCT03740893)

This phase 2a trial enrolled patients with neoadjuvant chemotherapy-resistant residual TNBC. The DDR/Anti-PD-L1 trial included two parts: post-neoadjuvant chemotherapy preoperative window of opportunity component and post-operative component. Experimental groups included monotherapy of AZD6738 (ATRi), olaparib, and durvalumab (anti- PD-L1 mAb). AZD6738 is an ATP competitive, orally bioavailable inhibitor of the ATR kinase. The biomarker changes in mean proliferation index, the proliferation gene expression signature, CD8+ TILs post anti-PD-L1 immunotherapy, and IFNγ+ signature is used as the primary endpoints of each group. The secondary outcome measurements include evaluation of the changes in phosphorylation of ATR and its downstream effectors (CHK1, γH2AX, CDC25, etc.). Changes in biomarkers of DDR (53BP1, RAD51, RPA, etc.) and adaptive and innate response (IFNγ, cGAS-STING pathway) are also measured.

#### 5.1.3. Monotherapy in Biomarker-Selected Patients

##### EMBRACA Trial (NCT01945775)

The trial selected 431 advanced and/or metastatic breast cancer patients with gBRCAm to compare the treatments of talazoparib (PARPi) versus physician’s-choice of chemotherapy. The patients must be selected based on an FDA-approved companion diagnostic for gBRCAm. Although talazoparib significantly improves progression-free survival (PFS) over chemotherapy [115], it does not show superior effect on patient’s OS [120].

##### OlympiAD Trial (NCT02000622)

The trial selected 302 metastatic breast cancer patients with gBRCAm to assess the efficacy and safety of olaparib monotherapy versus physician’s choice of chemotherapy (capecitabine, vinorelbine or eribulin). Among patients with HER2-negative metastatic breast cancer and gBRCAm, olaparib monotherapy provided a significant benefit over chemotherapy [117]. The olaparib has been approved by FDA for gBRCAm, HER2-negative metastatic breast cancer and ovary cancer [72,121,122].

##### NCT03428802

This trial evaluated the response rate of pembrolizumab (anti-PD-1 mAb) in advanced breast cancer or ovary cancer with genomic instability with POLE, POLD1 or BRCA mutations. The DNA polymerase δ/ε mediate the post-incision polymerization steps in BER, NER and MMR (Figure 2). POLE, POLD1 have been considered as predictive biomarkers for immunotherapy in many cancer types, including melanoma, colorectal, and endometrial cancers [123].

### 5.2. Colorectal Cancer (CRC)

The CRC is the third most common cancer and the second causes of cancer death in 2020 (1.93 million cases). The inactivation of MMR genes MSH2 and MLH1 by mutation or epigenetic silencing is an important cause of CRC [41]. Alterations of other MMR genes (MSH6, MSH1, PMS2, MLH3 and EXO1) and HRR genes (ATM, BRCA1, and Rad51) are also found in CRC [73]. These mutations cause dMMR in sporadic and hereditary CRC, in which abnormal lengths of dinucleotide repeat sequences, a representative of MSI, are found in a high frequency (~15%) [124]. These DDR features are good biomarkers for cancer immunotherapy and may be ideal targets of DDR inhibitors [125]. FDA has approved pembrolizumab to treat MSI-H or dMMR solid tumors including CRC [101,102,126]. Table 2 shows the clinical trials of ICIs and DDR-targeting therapy in CRC.

#### 5.2.1. Combination Therapy

##### DAPPER Trial (NCT03851614)

This trial enrolls patients with advanced pMMR CRC, pancreatic adenocarcinoma, or leiomyosarcoma to evaluate the changes in genomic and immune biomarkers in response to the combination treatment of durvalumab (anti PD-L1 mAb) with olaparib or cediranib (VEGFi).

##### NCT02484404

The trial enrolls 384 participants with advanced or recurrent ovarian, TNBC, lung, prostate and colorectal cancers. The immunotherapy regimens are durvalumab (anti PD-L1) in combination with olaparib (PARPi) and/or cediranib (anti VEGF). In prostate cancer patients, the toxicity of durvalumab plus olaparib is acceptable and the combination therapy is effective, particularly in men with DDR abnormalities. The early changes in circulating tumor cell counts and immune characteristics are associated with response [15]. The phase 1 results in ovary cancer show that the combination therapy is safe and tolerable [127].

#### 5.2.2. Monotherapy in Biomarker-Selected Patients

##### KEYNOTE-177 Trial (NCT02563002)

In total, 307 participants are selected with MSI-H or dMMR advanced colorectal carcinoma. They were randomly assigned to receive either pembrolizumab or the investigator’s choice. The FDA has approved pembrolizumab for patients with previously untreated unresectable or metastatic MSI-H or dMMR colorectal cancer [128].

##### NCT01876511

This study enrolls 113 participants, including CRC (MSI or MSS) and non-colorectal cancers (MSI). In addition to CRC. This important trial expanded to evaluate efficacy of PD-1 blockade (Pembrolizumab, MK-3475) in patients with advanced dMMR cancers across 12 different tumor types. The result of CRC has shown that the immune-related objective response rate and immune-related progression-free survival rate were 40% and 78%, respectively, for dMMR colorectal cancers and 0% and 11% for MMR–proficient (pMMR) colorectal cancers [42]. The efficacy and functional analysis are good enough to support the hypothesis that the large proportion of mutant neoantigens in dMMR cancers make them sensitive to immune checkpoint blockade, regardless of the origin of cancers. Functional analysis in a responding patient demonstrated rapid in vivo expansion of neoantigen-specific T cell clones that were reactive to cancer’s mutant neoantigen [129].

**Table 2:** Clinical trials of colorectal cancer using immunotherapy or synthetic lethality strategy.

Treatment	DDRi	Target	ICI	Target	TT/CT ^1^	Disease Setting	Biomarker	Trial ID	Phase	Comment/Reference
Combination	Olaparib	PARP	Durvalumab	PD-L1	Cediranib (VEGF)	CRC ^2^, PA ^3^, LMS ^4^	MSS ^5^, pMMR ^6^	NCT03851614	II	DAPPER trial
	Olaparib	PARP	Durvalumab	PD-L1	Cediranib (VEGF)	Solid tumors		NCT02484404	I/II	[15,127]
Monotherapy (ICI)			Pembrolizumab	PD-1		CRC, solid tumor	MSI ^7^-H or dMMR, TMB ^8^-H	NCT02563002	approved	KEYNOTE-177 trial
			Pembrolizumab	PD-1		CRC, MSI+ non-CRC	CRC (MSI or MSS)	NCT01876511	II	[42,129]
			Nivolumab Ipilimumab	PD-1 CTLA-4	Celecoxib (COX-2i)	mCRC ^9^	MSI, MSS	NCT03026140	II	NICHE trial [130]
			Avelumab	PD-L1		mCRC	MSI-H or POLEm ^10^	NCT03150706	II	
			Durvalumab	PD-L1		mCRC	MSI-H or POLEm	NCT03435107	II	
Monothreapy (DDRi)	AZD1775	WEE1			Irinotecan (Top1)	mCRC	RAS, BRAFm ^12^	NCT02906059	I	
	Niraparib	PARP			Panitumumab (EGFR)	aCRC ^11^ and mCRC	RasWT or MSI-H or MSS	NCT03983993	II	NIPAVect trial
	Olaparib	PARP			Temozolomide	aCRC	MGMT ^13^ promoter hypermethylation	NCT04166435	II	
	Adavosertib	WEE1				mCRC	RAS, TP53	FOCUS4-C	II	[44]

^1^ TT/CT = Target Therapy/Chemotherapy; ^2^ CRC = Colorectal Cancer; ^3^ PA = Pancreatic Adenocarcinoma; ^4^ LMS = Leiomyosarcoma; ^5^ MSS = Microsatellite Stability; ^6^ pMMR = Mismatch Repair Proficient; ^7^ MSI = Microsatellite Instability; ^8^ TMB = Tumor mutational burden; ^9^ mCRC = Metastatic Colorectal Cancer; ^10^ POLEm = POLE mutated; ^11^ aCRC = Adanced Colorectal Cancer; ^12^ BRAFm = BRAF mutations; ^13^ MGMT = Methylguanine methyltransferase.

##### NICHE Trial (NCT03026140)

This trial compares the neoadjuvant efficacies of nivolumab and ipilimumab between early stage patients with dMMR and pMMR. Pathological responses are observed in 100% dMMR tumors and in 27% pMMR tumors, which can be predicted by CD8+PD-1+ T cell infiltration in pMMR tumors. These data suggest that neoadjuvant immunotherapy is effective in a defined patient group [130].

##### NCT03150706 and NCT03435107

These trials select CRC patients according to POLE mutations, which have been reported in colorectal and various cancers [131]. This study suspects that patients with POLE mutations represent high TMB and will be sensitive to ICB therapy. The efficacies of the PD-L1 inhibitors avelumab and durvalumab will be evaluated in these trials.

##### NCT02906059

WEE1 kinase controls G2/M checkpoint. Inhibition of WEE1 will lead replication and mitotic catastrophe. Topoisomerase 1 (Top1) generates transient SSBs to resolve topological stresses during DNA replication. Inhibition of Top1 causes topoisomerase cleavage complex trapping. In replicating cells, these lesions result in replication fork arrest, collapse, and eventually DSBs in S phase, which is highly toxic to the cells. Combination of WEE1 and Top 1 inhibitors (AZD1775 and irinotecan) is tested in highly proliferated mCRC with RAS (KRAS or NRAS) or BRAF mutations. This phase 1 trial evaluates the safety and efficacy of this combination regimen in RAS/BRAF-mutated metastatic colorectal cancer patients.

##### NIPAVect Trial (NCT03983993)

This phase 2 trial selects CRC with wild-type RAS or MSI-H or metastatic MSS CRC. It evaluated the activity of the combination of niraparib (PARPi) with panitumumab (EGFRi).

##### NCT04166435

This phase 2 trial selects CRC patients with hypermethylated MGMT promoter. MGMT is a key gene that encodes for an enzyme that removes alkylated DNA and directly repairs. Hypermethylated promoter of MGMT cause deficient one-step direct repair system. Temozolomide (TMZ) is an oral alkylating prodrug which delivers a methyl group to purine bases of DNA (O^6^-guanine; N^7^-guanine and N^3^-adenine). The TMZ alone does not gain promising clinical benefit in CRC [132]. The trial will determine the efficacy of TMZ in combination with PARP inhibitor olaparib and characterize DNA methylation and gene expression profiles.

##### FOCUS4-C Trial

FOCUS4 is a phase 2 trial for biomarker-selected CRC. This study hypothesizes that aberrations in DNA replication in mCRC with RAS and TP53 mutations will sensitize tumors to WEE1 inhibitor (AZD1775, adavosertib). The improved PFS demonstrates that adavosertib is a potential well-tolerated therapeutic in RAS/TP53-mutated mCRC [44].

### 5.3. Pancreatic Cancer

Pancreatic cancer is one of the most malignant human cancers with the poorest prognosis. Whole-exome and whole-genome sequencing of PDAC have confirmed alterations in the critical driver genes in the majority of pancreatic cancers [49,133]. The mutations of KRAS and TP53 account for >90% and 40–75% of pancreatic cancer, respectively. The most common germline mutations are found in BRCA2 gene (1.4–7.4%). The other familiar susceptibility genes include ATM, PALB2, MLH1, MSH2, CDKN2A, and PRSS1. Somatic mutations of BRCA1, BRCA2 and PALB2 are ~14% in pancreatic cancer [134,135]. The DDR deficiency occurs in up to 24% pancreatic cancer [49,136]. These mutational landscapes provide implications for alternative therapeutic strategies beyond chemotherapy through targeting DDR pathways and immunotherapy. Table 3 lists clinical trials of ICB and DDR-targeting therapies in pancreatic cancer.

#### 5.3.1. Combination Therapy

##### DAPPER Trial (NCT03851614)

This phase 2 trial has been mentioned in CRC (Section 5.2.1. The goal (primary outcome) of this study is to measure the changes in genomic and immune biomarkers after the combination of durvalumab and olaparib or cediranib.

##### NCT04548752

This trial studies whether pembrolizumab plus olaparib works better than olaparib alone (standard of care) in patients with gBRCAm metastatic pancreatic cancer, which tests the concept of improved onco-immunology by combination of DDR targeting and ICIs.

##### Parpvax Trial (NCT03404960)

The phase 1b/2 trial enrolls 84 pancreatic cancer patients whose disease has not progressed on platinum-based therapy. The efficacies (PFS) of niraparib (PARPi) plus either ipilimumab or nivolumab will be measured.

#### 5.3.2. Monotherapy with DDR Inhibitors or ICIs

##### The Match Screening Trial (NCT02465060)

This phase 2 trial enrolls 6452 participants with progressive lymphomas or solid cancers including pancreatic cancer to examine the correlation between treatments and the corresponding matched genetic alterations in cancer tissues, which is named as “Molecular Analysis for Therapy Choice” (MATCH) trial. For example, the patients with dMMR (loss of MLH1 or MSH2 by IHC) receive nivolumab and the patients with BRCA1 or BRCA2 mutations are treated with adavosertib (WEE1 inhibitor) [137]. This trial has reported that 37.6% of specimens have actionable genetic alterations; however, less than 6% is found in pancreatic cancer.

#### 5.3.3. DDR Inhibitors in Combination with Chemotherapy or Radiotherapy

##### NCT04514497

This phase I trial investigates the side effects and the optimal dose of elimusertib (BAY 1895344, an orally available ATR kinase inhibitor) when given together with chemotherapy (irinotecan or topotecan, both are topoisomerase inhibitors). This trial also evaluates the changes in DDR gene expression patterns, such as the phosphorylation of histone H2AX and NBS1.

##### NCT04172532

The trial studies the side effects and best dose of peposertib (M3814) and to see how well it works when given together with radiotherapy in patients with pancreatic cancer. Peposertib is a potent DNA-PK inhibitor (DNA-PKi) in early clinical development. In acute leukemia, peposertib can enhance p53-dependent cytotoxicity in the presence of topoisomerase inhibitors or ionizing radiation [70].

##### NCT01296763

The trial enrolls patients whose pancreatic cancers have defects in the BRCA/Fanconi anemia (FANC) HRR pathway. The treatment agents are olaparib and a combination regimen of DNA damaging agents, irinotecan, cisplatin, mitomycin C (ICM). Durable clinical responses are observed in a subset of patients. Olaparib has substantial toxicity when combined with IC or ICM [138].

**Table 3:** Clinical trials of pancreatic cancer using immunotherapy or synthetic lethality strategy.

Treatment	DDRi	Target	ICI	Target	CT/RT	Disease Setting	Biomarker	Trial ID	Phase	Comment/Reference
Combination DDRi+ICi	Olaparib	PARP	Durvalumab	PD-L1	Cediranib (VEGFi)	PC ^1^, CRC ^2^,LMS ^3^	MSS ^4^	NCT03851614	II	DAPPER trial
	Olaparib	PARP	Pembrolizumab	PD-1		mPC ^5^	gBRCA1/2m	NCT04548752	II	
	Niraparib	PARP	Nivolumab	PD-1	After platinum-based CT	Progression Free PC		NCT03404960	Ib	Parpvax trial
			Ipilimumab	CTLA-4					II	
Molecular Match	-	-	Nivolumab	PD-1		PC, BC ^6^, CRC	MLH1, MSH2,	NCT02465060	II	[137]
	Adavosertib	WEE1	-	-			BRCA1/2			NCI match trail
Combination	Elimusertib	ATR			Irinotecan	aPC ^7^, SCLC ^8^ PD-NEC ^9^		NCT04514497	I	
DDRi+CT/RT	Elimusertib	ATR			Topotecan					
	Peposertib	DNA-PK			Hypofractionated RT	localized aPC		NCT04172532	I/II	[70]
	Olaparib	PARP			Irinotecan, Cisplatin, Mitomycin	aPC	BRCA/FANC, HRD	NCT01296763	I	[138]
	Veliparib	PARP			Cisplatin, Gemcitabine	mPC	BRCA/PALB2	NCT01585805	II	[139]
	Veliparib	PARP			FOLFOX ^10^	mPC	BRCA1/2, PALBB2, FANC	NCT01489865	I/II	[140]
	Rucaparib	PARP			Irinotecan Fluorouracil, Leucovorin	mPC, CRC	BRCA1/2, PALB2 (HRD ^11^)	NCT03337087	I/II	[141]
	Olaparib Selumetinib	PARP MEK			FOLFIRI ^12^	mPC	sBRCAm, KRAS or no mutation	NCT04348045		MAZEPPA trial
							
Monotherapy PARPi (Molecular Selection)	Olaparib	PARP				mPC	gBRCA1/2m	NCT02184195	approved	POLO trial. [142]
	Olaparib	PARP				PC, OC ^13^, BC Prostate Ca	gBRCA1/2m	NCT01078662	II	[143]
	Rucaparib	PARP				PC, EC ^14^, OC, etc.	BRCA1/2, PALB2, RAD51C, RAD51D, BARD1, BRIP1, FANCA, NBN, RAD51, RAD51B	NCT04171700	II	LODESTAR trial
	Niraparib	PARP				PC	BRCA1/2, PALB2, CHEK2 ATM	NCT03601923	II	

^1^ PC = Pancreatic Cancer; ^2^ CRC = Colorectal Cancer; ^3^ LMS = Leiomyosarcoma; ^4^ MSS = Microsatellite Stability; ^5^ mPC = Metastatic Pancreatic Cancer; ^6^ BC = Breast Cancer; ^7^ aPC = Advanced Pancreatic Cancer; ^8^ SCLC = Small Cell Lung Carcinoma; ^9^ PD-NEC = Poorly Differentiated Neuroendocrine Carcinoma; ^10^ FOLFOX-6 = 5-Fluorouracil Plus Oxaliplatin; ^11^ HRD = Homologous Recombination Deficiency; ^12^ FOLFIRI = Folinic Acid Plus Fluorouracil Plus Irinotecan; ^13^ OC = Ovary Cancer; ^14^ EC = Esophageal Cancer.

##### NCT01585805

This phase 2 trial evaluates the efficacy of veliparib (ABT-888, PARPi) in 16 patients with previously treated BRCA1/2- or PALB2-mutated pancreatic cancer. The results show no confirmed response, suggesting that development of other therapeutics are needed for the pancreatic cancer patients with BRCA1/2 or PALB2 mutations [139].

##### NCT01489865

This phase 1/2 trial tests the effectiveness of veliparib in combination with mFOLFOX-6 (modified 5-fluorouracil and oxaliplatin) for 64 patients with metastatic pancreatic cancer. The patient inclusion criteria include positive family history of breast or ovarian cancer or HRD mutations in the BRCA1, BRCA2, PALBB2, or FANC genes. The results suggest that combination of veliparib and FOLFOX is effective in patients harboring HRD mutations [140].

##### NCT03337087

This is a phase 1/2 trial to examine the safety and the best dose of irinotecan liposome (Nal-IRI) and rucaparib plus fluorouracil and leucovorin in the treatment of 110 patients with metastatic gastrointestinal malignancies. The selected pancreatic cancer patients with BRCA1/2 and PALB2 mutations and HRD are included in this study.

##### MAZEPPA Trial (NCT04348045)

This is an open-label, phase 2 study to assess the efficacy of a genomic-driven maintenance therapy in terms of PFS in 307 pancreatic cancer patients in three groups: olaparib for BRCAness (arm A), and randomization of durvalumab plus selumetinib (MEK inhibitor) [144] (arm B) or FOLFIRI (folinic acid, fluorouracil and irinotecan) chemotherapy (arm C) for those with KRAS mutation and negative of BRCAness.

#### 5.3.4. Monotherapy with PARP Inhibitor in Biomarker-Selected Patients

##### POLO Trial (NCT02184195)

This phase 3 trial tests the efficacy of olaparib monotherapy in 154 patients with gBRCAm metastatic pancreatic cancer whose disease has not progressed on first line platinum-based chemotherapy. The results show that olaparib increases PFS when compared with placebo [142]. In 2019, FDA has approved olaparib for the maintenance treatment of gBRCAm metastatic pancreatic cancer.

##### NCT01078662

This phase 2 trial evaluates the efficacy of olaparib in 299 patients with advanced breast, ovarian, prostate, and pancreatic cancers harboring BRCA1 and/or BRCA2 mutations. The results in gBRCAm ovary cancer patients show notable antitumor activity and durable responses [121,143].

##### Lodestar Trial (NCT04171700)

This phase 2 trial enrolls 220 patients with HRD solid tumors to evaluate the efficacy of rucaparib. The patients are assigned into two arms according to mutations in BRCA1, BRCA2, PALB2, RAD51C, RAD51D (Cohort A) and mutations in BARD1, BRIP1, FANCA, NBN, RAD51 or RAD51B (Cohort B).

##### NCT03601923

This phase 2 trial evaluates the safety and effectiveness of niraparib in 32 patients with pancreatic cancer harboring HRD.

## 6. Discussion and Future Perspectives

Although the targeting of DDR and immune checkpoint hold onto the hope for a cure, this therapeutic strategy is effective only on a subgroup of patients with specific DDR and immune biomarkers, such as HRD, TMB-high, MSI-H, dMMR, and expression of immune checkpoint molecules [22,24,25]. Therefore, appropriate biomarker examination is required to guide patient selection. At present, there is still no consensus on the methods of biomarker examination for proper patient selection in this setting. Although FDA has approved the two HRD diagnostic tests, myChoice CDx and FoundationOne CDx, some problems remain to be solved, such as the determination of proper thresholds for HRD scores, which will influence the classification of patients with or without HRD phenotype [145]. Moreover, new and convenient methodologies that cover specific HRR gene panels depending on different settings are also required.

In addition to genetic alterations that are detected by the two HRD diagnostic tests, HRD phenotype can be induced by gene silencing through epigenetic mechanisms or by dysregulation of gene function. In line with this notion, many studies have found that some HRD-negative patients also respond to PARP inhibitors [60,146,147]. The reason underlying this responsiveness in HRD-negative patients is still unclear. It is worthy to investigate the genetic, epigenetic, and functional statuses of DDR and immune genes in the tumors of these “exceptional responders”, which may shed light on new directions for the development of cancer therapy [148].

Notably, some studies have shown that targeting oncogenic pathways or epigenetic modifiers can improve the efficacies of DDR and immune checkpoint inhibitors [13,14,149]. For example, targeting PI3K leads to the downregulation of BRCA1/2 and sensitizes BRCA-proficient TNBC cells to PARP inhibitors [150]. Inhibition of DNA methyltransferase results in HRD phenotype and sensitizes BRCA-proficient NSCLC cells to PARP inhibitors [151]. These studies demonstrate that PARP inhibitors are effective in HRD-negative tumors by concurrent inhibition of oncogenic pathway or epigenetic modifier, highlighting the usefulness of PARP inhibitors in HRD-negative cancer patients. Targeting epigenetic modifiers, such as the histone demethylase 1 (LSD1), can re-activate the expression of endogenous retrovirus, which triggers type I interferon response and enhances the efficacy of ICIs [149,152]. Therefore, combination targeting of DDR, immune checkpoint, oncogenic pathways or epigenetic modifiers can efficiently inhibit cancer cells; however, treatment toxicity may be increased.

Cancer cells are dynamically evolved, especially under therapeutic pressure, which leads to treatment-resistant clones. Indeed, secondary mutations of BRCA1/2 genes that restore HRR function are found in patient’s circulating cell-free DNA [153]. In advanced cancers with obvious heterogeneity, some tumor cells may exhibit intrinsic resistance to therapy. Both acquired and intrinsic resistant events result in treatment failure and cancer relapse [6,94]. Thus, additional biomarkers and therapeutics for resistant tumor cells are required for the comprehensive treatment of cancer patients.

## 7. Conclusions

Alterations of DDR genes that cause dMMR and HRD are frequently observed in cancers. Although these genetic alterations may promote cancer development, they can also serve as biomarkers for the selection of suitable patients for specific therapeutics, including ICB therapy. In addition, DDR alterations can be good targets for therapy. Investigations of the interplay of DDR-targeting and ICB therapies can provide more effective treatment options for cancer patients.

## Data Availability

Not applicable.

## References

[1] Bray F., Ferlay J., Soerjomataram I., Siegel R.L., Torre L.A., Jemal A. (2018). Global cancer statistics 2018: GLOBOCAN estimates of incidence and mortality worldwide for 36 cancers in 185 countries. CA Cancer J. Clin..

[2] Hanahan D., Weinberg R.A. (2000). The Hallmarks of Cancer. Cell.

[3] Hanahan D., Weinberg R.A. (2011). Hallmarks of cancer: The next generation. Cell.

[4] Khaddour K., Jonna S., Deneka A., Patel J., Abazeed M., Golemis E., Borghaei H., Boumber Y. (2021). Targeting the Epidermal Growth Factor Receptor in EGFR-Mutated Lung Cancer: Current and Emerging Therapies. Cancers.

[5] Le X., Nilsson M., Goldman J., Reck M., Nakagawa K., Kato T., Ares L.P., Frimodt-Moller B., Wolff K., Visseren-Grul C. (2020). Dual EGFR-VEGF Pathway Inhibition: A Promising Strategy for Patients with EGFR-Mutant NSCLC. J. Thorac. Oncol..

[6] Sabnis A.J., Bivona T.G. (2019). Principles of Resistance to Targeted Cancer Therapy: Lessons from Basic and Translational Cancer Biology. Trends Mol. Med..

[7] Kopetz S., Grothey A., Yaeger R., Van Cutsem E., Desai J., Yoshino T., Wasan H., Ciardiello F., Loupakis F., Hong Y.S. (2019). Encorafenib, Binimetinib, and Cetuximab in BRAF V600E–Mutated Colorectal Cancer. N. Engl. J. Med..

[8] Wheeler D.L., Dunn E.F., Harari P.M. (2010). Understanding resistance to EGFR inhibitors—impact on future treatment strategies. Nat. Rev. Clin. Oncol..

[9] Hoeijmakers J.H. (2001). Genome maintenance mechanisms for preventing cancer. Nature.

[10] Bartkova J., Hořejší Z., Koed K., Krämer A., Tort F., Zieger K., Guldberg P., Sehested M., Nesland J.M., Lukas C. (2005). DNA damage response as a candidate anti-cancer barrier in early human tumorigenesis. Nature.

[11] Gourley C., Balmaña J., Ledermann J.A., Serra V., Dent R., Loibl S., Pujade-Lauraine E., Boulton S.J. (2019). Moving From Poly (ADP-Ribose) Polymerase Inhibition to Targeting DNA Repair and DNA Damage Response in Cancer Therapy. J. Clin. Oncol..

[12] Dickson K.-A., Xie T., Evenhuis C., Ma Y., Marsh D.J. (2021). PARP Inhibitors Display Differential Efficacy in Models of *BRCA* Mutant High-Grade Serous Ovarian Cancer. Int. J. Mol. Sci..

[13] Pilié P.G., Tang C., Mills G.B., Yap T.A. (2018). State-of-the-art strategies for targeting the DNA damage response in cancer. Nat. Rev. Clin. Oncol..

[14] Chabanon R.M., Rouanne M., Lord C.J., Soria J.-C., Pasero P., Postel-Vinay S. (2021). Targeting the DNA damage response in immuno-oncology: Developments and opportunities. Nat. Cancer.

[15] Karzai F., VanderWeele D., Madan R.A., Owens H., Cordes L.M., Hankin A., Couvillon A., Nichols E., Bilusic M., Beshiri M. (2018). Activity of durvalumab plus olaparib in metastatic castration-resistant prostate cancer in men with and without DNA damage repair mutations. J. Immunother. Cancer.

[16] Rizzo A., Brandi G. (2021). Biochemical predictors of response to immune checkpoint inhibitors in unresectable hepatocellular carcinoma. Cancer Treat. Res. Commun..

[17] Ricci A.D., Rizzo A., Brandi G. (2020). The DNA damage repair (DDR) pathway in biliary tract cancer (BTC): A new Pandora’s box?. ESMO Open.

[18] Blackford A.N., Jackson S.P. (2017). ATM, ATR, and DNA-PK: The Trinity at the Heart of the DNA Damage Response. Mol. Cell.

[19] Tomimatsu N., Tahimic C.G.T., Otsuki A., Burma S., Fukuhara A., Sato K., Shiota G., Oshimura M., Chen D.J., Kurimasa A. (2007). Ku70/80 Modulates ATM and ATR Signaling Pathways in Response to DNA Double Strand Breaks. J. Biol. Chem..

[20] Falck J., Coates J., Jackson S.P. (2005). Conserved modes of recruitment of ATM, ATR and DNA-PKcs to sites of DNA damage. Nature.

[21] Roos W.P., Thomas A.D., Kaina B. (2016). DNA damage and the balance between survival and death in cancer biology. Nat. Rev. Cancer.

[22] Mouw K.W., Goldberg M.S., Konstantinopoulos P.A., D’Andrea A.D. (2017). DNA Damage and Repair Biomarkers of Immunotherapy Response. Cancer Discov..

[23] Zhang J., Shih D.J.H., Lin S.-Y. (2020). Role of DNA repair defects in predicting immunotherapy response. Biomark. Res..

[24] Jiang M., Jia K., Wang L., Li W., Chen B., Liu Y., Wang H., Zhao S., He Y., Zhou C. (2021). Alterations of DNA damage response pathway: Biomarker and therapeutic strategy for cancer immunotherapy. Acta Pharm. Sin. B.

[25] Yang C., Zhang Z., Tang X., Zhang X., Chen Y., Hu T., Zhang H., Guan M., Zhang X., Wu Z. (2021). Pan-cancer analysis reveals homologous recombination deficiency score as a predictive marker for immunotherapy responders. Hum. Cell.

[26] Pearl L.H., Schierz A.C., Ward S.E., Al-Lazikani B., Pearl F.M. (2015). Therapeutic opportunities within the DNA damage response. Nat. Rev. Cancer.

[27] Curtin N.J. (2012). DNA repair dysregulation from cancer driver to therapeutic target. Nat. Cancer.

[28] Weller M., Stupp R., Reifenberger G., Brandes A.A., Bent M.J.V.D., Wick W., Hegi M.E. (2010). MGMT promoter methylation in malignant gliomas: Ready for personalized medicine?. Nat. Rev. Neurol..

[29] Lord C.J., Ashworth A. (2012). The DNA damage response and cancer therapy. Nature.

[30] Cressey D. (2015). DNA repair sleuths win chemistry Nobel. Nature.

[31] Helleday T., Petermann E., Lundin C., Hodgson B., Sharma R.A. (2008). DNA repair pathways as targets for cancer therapy. Nat. Rev. Cancer.

[32] Marteijn J.A., Lans H., Vermeulen W., Hoeijmakers J.H. (2014). Understanding nucleotide excision repair and its roles in cancer and ageing. Nat. Rev. Mol. Cell Biol..

[33] Okimoto T., Tsubata Y., Tanino R., Nakao M., Hotta T., Hamaguchi M., Hamaguchi S., Araki A., Isobe T. (2021). ERCC1 Is a Predictive Biomarker for Non-small Cell Lung Cancer But Is Antibody-dependent. Anticancer Res..

[34] Olaussen K.A., Dunant A., Fouret P., Brambilla E., Andre F., Haddad V., Taranchon E., Filipits M., Pirker R., Popper H.H. (2006). DNA Repair by ERCC1 in Non–Small-Cell Lung Cancer and Cisplatin-Based Adjuvant Chemotherapy. N. Engl. J. Med..

[35] Chaudhuri A.R., Nussenzweig A. (2017). The multifaceted roles of PARP1 in DNA repair and chromatin remodelling. Nat. Rev. Mol. Cell Biol..

[36] Rouleau M., Patel A., Hendzel M.J., Kaufmann S.H., Poirier G.G. (2010). PARP inhibition: PARP1 and beyond. Nat. Cancer.

[37] Pommier Y., O’Connor M.J., de Bono J. (2016). Laying a trap to kill cancer cells: PARP inhibitors and their mechanisms of action. Sci. Transl. Med..

[38] Lord C.J., Ashworth A. (2017). PARP inhibitors: Synthetic lethality in the clinic. Science.

[39] Livraghi L., Garber J.E. (2015). PARP inhibitors in the management of breast cancer: Current data and future prospects. BMC Med..

[40] Jiricny J. (2006). The multifaceted mismatch-repair system. Nat. Rev. Mol. Cell Biol..

[41] Kim T.-M., Laird P.W., Park P.J. (2013). The Landscape of Microsatellite Instability in Colorectal and Endometrial Cancer Genomes. Cell.

[42] Le D.T., Uram J.N., Wang H., Bartlett B.R., Kemberling H., Eyring A.D., Skora A.D., Luber B.S., Azad N.S., Laheru D. (2015). PD-1 Blockade in Tumors with Mismatch-Repair Deficiency. N. Engl. J. Med..

[43] Elbæk C.R., Petrosius V., Sørensen C.S. (2020). WEE1 kinase limits CDK activities to safeguard DNA replication and mitotic entry. Mutat. Res. Mol. Mech. Mutagen..

[44] Seligmann J.F., Fisher D.J., Brown L.C., Adams R.A., Graham J., Quirke P., Richman S.D., Butler R., Domingo E., Blake A. (2021). Inhibition of WEE1 Is Effective in TP53- and RAS-Mutant Metastatic Colorectal Cancer: A Randomized Trial (FOCUS4-C) Comparing Adavosertib (AZD1775) With Active Monitoring. J. Clin. Oncol..

[45] Zhao B., Rothenberg E., Ramsden D.A., Lieber M.R. (2020). The molecular basis and disease relevance of non-homologous DNA end joining. Nat. Rev. Mol. Cell Biol..

[46] Isono M., Niimi A., Oike T., Hagiwara Y., Sato H., Sekine R., Yoshida Y., Isobe S.-Y., Obuse C., Nishi R. (2017). BRCA1 Directs the Repair Pathway to Homologous Recombination by Promoting 53BP1 Dephosphorylation. Cell Rep..

[47] Prakash R., Zhang Y., Feng W., Jasin M. (2015). Homologous Recombination and Human Health: The Roles of BRCA1, BRCA2, and Associated Proteins. Cold Spring Harb. Perspect. Biol..

[48] Welcsh P.L. (2001). BRCA1 and BRCA2 and the genetics of breast and ovarian cancer. Hum. Mol. Genet..

[49] Park W., Chen J., Chou J.F., Varghese A.M., Yu K.H., Wong W., Capanu M., Balachandran V., McIntyre C.A., El Dika I. (2020). Genomic Methods Identify Homologous Recombination Deficiency in Pancreas Adenocarcinoma and Optimize Treatment Selection. Clin. Cancer Res..

[50] Tomasova K., Cumova A., Seborova K., Horák J., Koucka K., Vodickova L., Vaclavikova R., Vodicka P. (2020). DNA Repair and Ovarian Carcinogenesis: Impact on Risk, Prognosis and Therapy Outcome. Cancers.

[51] Clague J., Wilhoite G., Adamson A., Bailis A., Weitzel J.N., Neuhausen S.L. (2011). RAD51C Germline Mutations in Breast and Ovarian Cancer Cases from High-Risk Families. PLoS ONE.

[52] Jones S., Hruban R.H., Kamiyama M., Borges M., Zhang X., Parsons D.W., Lin J.C.-H., Palmisano E., Brune K., Jaffee E.M. (2009). Exomic Sequencing Identifies PALB2 as a Pancreatic Cancer Susceptibility Gene. Science.

[53] Tischkowitz M.D., Sabbaghian N., Hamel N., Borgida A., Rosner C., Taherian N., Srivastava A., Holter S., Rothenmund H., Ghadirian P. (2009). Analysis of the Gene Coding for the BRCA2-Interacting Protein PALB2 in Familial and Sporadic Pancreatic Cancer. Gastroenterology.

[54] Slater E., Langer P., Niemczyk E., Strauch K., Butler J., Habbe N., Neoptolemos J., Greenhalf W., Bartsch D. (2010). PALB2 mutations in European familial pancreatic cancer families. Clin. Genet..

[55] Macedo G.S., Alemar B., Ashton-Prolla P. (2019). Reviewing the characteristics of BRCA and PALB2-related cancers in the precision medicine era. Genet. Mol. Biol..

[56] Bryant H.E., Schultz N., Thomas H.D., Parker K.M., Flower D., Lopez E., Kyle S., Meuth M., Curtin N.J., Helleday T. (2005). Specific killing of BRCA2-deficient tumours with inhibitors of poly(ADP-ribose) polymerase. Nature.

[57] Farmer H., McCabe N., Lord C.J., Tutt A.N.J., Johnson D.A., Richardson T.B., Santarosa M., Dillon K.J., Hickson I., Knights C. (2005). Targeting the DNA repair defect in BRCA mutant cells as a therapeutic strategy. Nature.

[58] Mateo J., Lord C.J., Serra V., Tutt A., Balmaña J., Castroviejo-Bermejo M., Cruz C., Oaknin A., Kaye S.B., de Bono J.S. (2019). A decade of clinical development of PARP inhibitors in perspective. Ann. Oncol..

[59] Byrum A., Vindigni A., Mosammaparast N. (2019). Defining and Modulating ‘BRCAness’. Trends Cell Biol..

[60] Ray-Coquard I., Pautier P., Pignata S., Pérol D., González-Martín A., Berger R., Fujiwara K., Vergote I., Colombo N., Mäenpää J. (2019). Olaparib plus Bevacizumab as First-Line Maintenance in Ovarian Cancer. N. Engl. J. Med..

[61] Mateo J., Carreira S., Sandhu S., Miranda S., Mossop H., Perez-Lopez R., Nava Rodrigues D., Robinson D., Omlin A., Tunariu N. (2015). DNA-Repair Defects and Olaparib in Metastatic Prostate Cancer. N. Engl. J. Med..

[62] Turner N., Tutt A., Ashworth A. (2004). Hallmarks of ‘BRCAness’ in sporadic cancers. Nat. Cancer.

[63] Fong P.C., Boss D.S., Yap T.A., Tutt A., Wu P., Mergui-Roelvink M., Mortimer P., Swaisland H., Lau A., O’Connor M.J. (2009). Inhibition of Poly(ADP-Ribose) Polymerase in Tumors fromBRCAMutation Carriers. N. Engl. J. Med..

[64] Hosoya N., Miyagawa K. (2014). Targeting DNA damage response in cancer therapy. Cancer Sci..

[65] Kim G., Ison G., McKee A.E., Zhang H., Tang S., Gwise T., Sridhara R., Lee E., Tzou A., Philip R. (2015). FDA Approval Summary: Olaparib Monotherapy in Patients with Deleterious Germline BRCA-Mutated Advanced Ovarian Cancer Treated with Three or More Lines of Chemotherapy. Clin. Cancer Res..

[66] LaFargue C., Molin G.Z.D., Sood A.K., Coleman R.L. (2019). Exploring and comparing adverse events between PARP inhibitors. Lancet Oncol..

[67] Sun C., Yin J., Fang Y., Chen J., Jeong K.J., Chen X., Vellano C.P., Ju Z., Zhao W., Zhang D. (2018). BRD4 Inhibition Is Synthetic Lethal with PARP Inhibitors through the Induction of Homologous Recombination Deficiency. Cancer Cell.

[68] Siddiqui A., Tumiati M., Joko A., Sandholm J., Roering P., Aakko S., Vainionpää R., Kaipio K., Huhtinen K., Kauppi L. (2021). Targeting DNA Homologous Repair Proficiency With Concomitant Topoisomerase II and c-Abl Inhibition. Front. Oncol..

[69] Lloyd R.L., Wijnhoven P.W.G., Ramos-Montoya A., Wilson Z., Illuzzi G., Falenta K., Jones G.N., James N., Chabbert C.D., Stott J. (2020). Combined PARP and ATR inhibition potentiates genome instability and cell death in ATM-deficient cancer cells. Oncogene.

[70] Haines E., Nishida Y., Carr M.I., Montoya R.H., Ostermann L.B., Zhang W., Zenke F.T., Blaukat A., Andreeff M., Vassilev L.T. (2021). DNA-PK inhibitor peposertib enhances p53-dependent cytotoxicity of DNA double-strand break inducing therapy in acute leukemia. Sci. Rep..

[71] McLornan D.P., List A., Mufti G.J. (2014). Applying Synthetic Lethality for the Selective Targeting of Cancer. N. Engl. J. Med..

[72] Arora S., Balasubramaniam S., Zhang H., Berman T., Narayan P., Suzman D., Bloomquist E., Tang S., Gong Y., Sridhara R. (2020). FDA Approval Summary: Olaparib Monotherapy or in Combination with Bevacizumab for the Maintenance Treatment of Patients with Advanced Ovarian Cancer. Oncologist.

[73] Mauri G., Arena S., Siena S., Bardelli A., Sartore-Bianchi A. (2020). The DNA damage response pathway as a land of therapeutic opportunities for colorectal cancer. Ann. Oncol..

[74] Das S., Cardin D. (2020). Targeting DNA Damage Repair Pathways in Pancreatic Adenocarcinoma. Curr. Treat. Options Oncol..

[75] Yu L., Liu P. (2021). Cytosolic DNA sensing by cGAS: Regulation, function, and human diseases. Signal Transduct. Target. Ther..

[76] Wang H., Hu S., Chen X., Shi H., Chen C., Sun L., Chen Z.J. (2017). cGAS is essential for the antitumor effect of immune checkpoint blockade. Proc. Natl. Acad. Sci. USA.

[77] Ishikawa H., Barber G.N. (2008). STING is an endoplasmic reticulum adaptor that facilitates innate immune signalling. Nature.

[78] Sun L., Wu J., Du F., Chen X., Chen Z.J. (2013). Cyclic GMP-AMP Synthase Is a Cytosolic DNA Sensor That Activates the Type I Interferon Pathway. Science.

[79] Parker B., Rautela B.S.P.J., Hertzog P. (2016). Antitumour actions of interferons: Implications for cancer therapy. Nat. Cancer.

[80] Pilger D., Seymour L.W., Jackson S.P. (2021). Interfaces between cellular responses to DNA damage and cancer immunotherapy. Genes Dev..

[81] Garcia-Diaz A., Shin D.S., Moreno B.H., Saco J., Escuin-Ordinas H., Rodriguez G.A., Zaretsky J.M., Sun L., Hugo W., Wang X. (2017). Interferon Receptor Signaling Pathways Regulating PD-L1 and PD-L2 Expression. Cell Rep..

[82] Lu C., Guan J., Lu S., Jin Q., Rousseau B., Lu T., Stephens D., Zhang H., Zhu J., Yang M. (2020). DNA Sensing in Mismatch Repair-Deficient Tumor Cells Is Essential for Anti-tumor Immunity. Cancer Cell.

[83] Deng L., Liang H., Xu M., Yang X., Burnette B., Arina A., Li X.-D., Mauceri H., Beckett M., Darga T. (2014). STING-Dependent Cytosolic DNA Sensing Promotes Radiation-Induced Type I Interferon-Dependent Antitumor Immunity in Immunogenic Tumors. Immunity.

[84] Storozynsky Q., Hitt M.M. (2020). The Impact of Radiation-Induced DNA Damage on cGAS-STING-Mediated Immune Responses to Cancer. Int. J. Mol. Sci..

[85] Cao X., Liang Y., Hu Z., Li H., Yang J., Hsu E.J., Zhu J., Zhou J., Fu Y.-X. (2021). Next generation of tumor-activating type I IFN enhances anti-tumor immune responses to overcome therapy resistance. Nat. Commun..

[86] Kwon J., Bakhoum S.F. (2020). The Cytosolic DNA-Sensing cGAS–STING Pathway in Cancer. Cancer Discov..

[87] Zhu Y., An X., Zhang X., Qiao Y., Zheng T., Li X. (2019). STING: A master regulator in the cancer-immunity cycle. Mol. Cancer.

[88] Buchbinder E.I., Desai A. (2016). CTLA-4 and PD-1 Pathways: Similarities, Differences, and Implications of Their Inhibition. Am. J. Clin. Oncol. Cancer Clin. Trials.

[89] Linsley P.S., Brady W., Urnes M., Grosmaire L.S., Damle N.K., Ledbetter J.A. (1991). CTLA-4 is a second receptor for the B cell activation antigen B7. J. Exp. Med..

[90] Krummel M.F., Allison J.P. (1995). CD28 and CTLA-4 have opposing effects on the response of T cells to stimulation. J. Exp. Med..

[91] Lipson E.J., Drake C.G. (2011). Ipilimumab: An Anti-CTLA-4 Antibody for Metastatic Melanoma. Clin. Cancer Res..

[92] Larkin J., Chiarion-Sileni V., Gonzalez R., Grob J.-J., Rutkowski P., Lao C.D., Cowey C.L., Schadendorf D., Wagstaff J., Dummer R. (2019). Five-Year Survival with Combined Nivolumab and Ipilimumab in Advanced Melanoma. N. Engl. J. Med..

[93] Sharma P., Hu-Lieskovan S., Wargo J.A., Ribas A. (2017). Primary, Adaptive, and Acquired Resistance to Cancer Immunotherapy. Cell.

[94] Jenkins R.W., Barbie D.A., Flaherty K.T. (2018). Mechanisms of resistance to immune checkpoint inhibitors. Br. J. Cancer.

[95] Lhuillier C., Rudqvist N.-P., Elemento O., Formenti S.C., DeMaria S. (2019). Radiation therapy and anti-tumor immunity: Exposing immunogenic mutations to the immune system. Genome Med..

[96] Lim J.Y.H., Gerber S.A., Murphy S.P., Lord E.M. (2014). Type I interferons induced by radiation therapy mediate recruitment and effector function of CD8+ T cells. Cancer Immunol. Immunother..

[97] Jiang M., Chen P., Wang L., Li W., Chen B., Liu Y., Wang H., Zhao S., Ye L., He Y. (2020). cGAS-STING, an important pathway in cancer immunotherapy. J. Hematol. Oncol..

[98] Li A., Yi M., Qin S., Song Y., Chu Q., Wu K. (2019). Activating cGAS-STING pathway for the optimal effect of cancer immunotherapy. J. Hematol. Oncol..

[99] Aval L.M., Pease J.E., Sharma R., Pinato D.J. (2020). Challenges and Opportunities in the Clinical Development of STING Agonists for Cancer Immunotherapy. J. Clin. Med..

[100] Wang Y., Zheng K., Xiong H., Huang Y., Chen X., Zhou Y., Qin W., Su J., Chen R., Qiu H. (2021). PARP Inhibitor Upregulates PD-L1 Expression and Provides a New Combination Therapy in Pancreatic Cancer. Front. Immunol..

[101] Lemery S., Keegan P., Pazdur R. (2017). First FDA Approval Agnostic of Cancer Site—When a Biomarker Defines the Indication. N. Engl. J. Med..

[102] Prasad V., Kaestner V., Mailankody S. (2018). Cancer Drugs Approved Based on Biomarkers and Not Tumor Type—FDA Approval of Pembrolizumab for Mismatch Repair-Deficient Solid Cancers. JAMA Oncol..

[103] Sen T., Rodriguez B.L., Chen L., Della Corte C.M., Morikawa N., Fujimoto J., Cristea S., Nguyen T., Diao L., Li L. (2019). Targeting DNA Damage Response Promotes Antitumor Immunity through STING-Mediated T-cell Activation in Small Cell Lung Cancer. Cancer Discov..

[104] Dufour J.H., Dziejman M., Liu M.T., Leung J.H., Lane T.E., Luster A.D. (2002). IFN-γ-Inducible Protein 10 (IP-10; CXCL10)-Deficient Mice Reveal a Role for IP-10 in Effector T Cell Generation and Trafficking. J. Immunol..

[105] Shi Z., Shen J., Qiu J., Zhao Q., Hua K., Wang H. (2021). CXCL10 potentiates immune checkpoint blockade therapy in homologous recombination-deficient tumors. Theranostics.

[106] Reisländer T., Groelly F.J., Tarsounas M. (2020). DNA Damage and Cancer Immunotherapy: A STING in the Tale. Mol. Cell.

[107] Gong E.A.Z., Yang Y., Zhang J., Guo W. (2021). Evaluation of 30 DNA damage response and 6 mismatch repair gene mutations as biomarkers for immunotherapy outcomes across multiple solid tumor types. Cancer Biol. Med..

[108] Watkins J.A., Irshad S., Grigoriadis A., Tutt A.N.J. (2014). Genomic scars as biomarkers of homologous recombination deficiency and drug response in breast and ovarian cancers. Breast Cancer Res..

[109] Domchek S.M., Postel-Vinay S., Im S.-A., Park Y.H., Delord J.-P., Italiano A., Alexandre J., You B., Bastian S., Krebs M.G. (2020). Olaparib and durvalumab in patients with germline BRCA-mutated metastatic breast cancer (MEDIOLA): An open-label, multicentre, phase 1/2, basket study. Lancet Oncol..

[110] Vinayak S., Tolaney S.M., Schwartzberg L., Mita M., McCann G., Tan A.R., Wahner-Hendrickson A.E., Forero A., Anders C., Wulf G.M. (2019). Open-label Clinical Trial of Niraparib Combined With Pembrolizumab for Treatment of Advanced or Metastatic Triple-Negative Breast Cancer. JAMA Oncol..

[111] Konstantinopoulos P.A., Waggoner S., Vidal G.A., Mita M., Moroney J.W., Holloway R., Van Le L., Sachdev J.C., Chapman-Davis E., Colon-Otero G. (2019). Single-Arm Phases 1 and 2 Trial of Niraparib in Combination With Pembrolizumab in Patients With Recurrent Platinum-Resistant Ovarian Carcinoma. JAMA Oncol..

[112] Juliá E.P., Amante A., Pampena M.B., Mordoh J., Levy E.M. (2018). Avelumab, an IgG1 anti-PD-L1 Immune Checkpoint Inhibitor, Triggers NK Cell-Mediated Cytotoxicity and Cytokine Production Against Triple Negative Breast Cancer Cells. Front. Immunol..

[113] Lorusso P., Pilat M.J.P., Santa-Maria C.A., Connolly R.M., Roesch E.E., Afghahi A., Han H.S., Nanda R., Wulf G.M., Assad H. (2020). Trial in progress: A phase II open-label, randomized study of PARP inhibition (olaparib) either alone or in combination with anti-PD-L1 therapy (atezolizumab) in homologous DNA repair (HDR) deficient, locally advanced or metastatic non-HER2-positive breast cancer. J. Clin. Oncol..

[114] Schmid P., Rugo H.S., Adams S., Schneeweiss A., Barrios C.H., Iwata H., Diéras V., Henschel V., Molinero L., Chui S.Y. (2020). Atezolizumab plus nab-paclitaxel as first-line treatment for unresectable, locally advanced or metastatic triple-negative breast cancer (IMpassion130): Updated efficacy results from a randomised, double-blind, placebo-controlled, phase 3 trial. Lancet Oncol..

[115] Litton J.K., Rugo H.S., Ettl J., Hurvitz S.A., Gonçalves A., Lee K.-H., Fehrenbacher L., Yerushalmi R., Mina L.A., Martin M. (2018). Talazoparib in Patients with Advanced Breast Cancer and a Germline BRCA Mutation. N. Engl. J. Med..

[116] Ettl J., Quek R., Lee K.-H., Rugo H., Hurvitz S., Gonçalves A., Fehrenbacher L., Yerushalmi R., Mina L., Martin M. (2018). Quality of life with talazoparib versus physician’s choice of chemotherapy in patients with advanced breast cancer and germline BRCA1/2 mutation: Patient-reported outcomes from the EMBRACA phase III trial. Ann. Oncol..

[117] Robson M., Im S.A., Senkus E., Xu B., Domchek S.M., Masuda N., Delaloge S., Li W., Tung N., Armstrong A. (2017). Olaparib for Metastatic Breast Cancer in Patients with a Germline BRCA Mutation. N. Engl. J. Med..

[118] Adams S., Diéras V., Barrios C., Winer E., Schneeweiss A., Iwata H., Loi S., Patel S., Henschel V., Chui S. (2020). Patient-reported outcomes from the phase III IMpassion130 trial of atezolizumab plus nab-paclitaxel in metastatic triple-negative breast cancer. Ann. Oncol..

[119] Wang H., Ma H., Sové R.J., Emens L.A., Popel A.S. (2021). Quantitative systems pharmacology model predictions for efficacy of atezolizumab and nab-paclitaxel in triple-negative breast cancer. J. Immunother. Cancer.

[120] Litton J., Hurvitz S., Mina L., Rugo H., Lee K.-H., Gonçalves A., Diab S., Woodward N., Goodwin A., Yerushalmi R. (2020). Talazoparib versus chemotherapy in patients with germline BRCA1/2-mutated HER2-negative advanced breast cancer: Final overall survival results from the EMBRACA trial. Ann. Oncol..

[121] Domchek S.M., Aghajanian C., Shapira-Frommer R., Schmutzler R.K., Audeh M.W., Friedlander M., Balmaña J., Mitchell G., Fried G., Stemmer S.M. (2015). Efficacy and safety of olaparib monotherapy in germline BRCA1/2 mutation carriers with advanced ovarian cancer and three or more lines of prior therapy. Gynecol. Oncol..

[122] Robson M.E., Tung N., Conte P., Im S.-A., Senkus E., Xu B., Masuda N., Delaloge S., Li W., Armstrong A. (2019). OlympiAD final overall survival and tolerability results: Olaparib versus chemotherapy treatment of physician’s choice in patients with a germline BRCA mutation and HER2-negative metastatic breast cancer. Ann. Oncol..

[123] Wang F., Zhao Q., Wang Y.-N., Jin Y., He M.-M., Liu Z.-X., Xu R.-H. (2019). Evaluation of POLE and POLD1 Mutations as Biomarkers for Immunotherapy Outcomes across Multiple Cancer Types. JAMA Oncol..

[124] Boland C.R., Goel A. (2010). Microsatellite instability in colorectal cancer. Gastroenterology.

[125] Seyedin S.N., Hasibuzzaman M., Pham V., Petronek M.S., Callaghan C., Kalen A.L., Mapuskar K.A., Mott S.L., Spitz D.R., Allen B.G. (2020). Combination Therapy with Radiation and PARP Inhibition Enhances Responsiveness to Anti-PD-1 Therapy in Colorectal Tumor Models. Int. J. Radiat. Oncol..

[126] Ganesh K., Stadler Z.K., Cercek A., Mendelsohn R.B., Shia J., Segal N.H., Diaz L.A. (2019). Immunotherapy in colorectal cancer: Rationale, challenges and potential. Nat. Rev. Gastroenterol. Hepatol..

[127] Zimmer A.S., Nichols E., Cimino-Mathews A., Peer C., Cao L., Lee M.-J., Kohn E.C., Annunziata C.M., Lipkowitz S., Trepel J.B. (2019). A phase I study of the PD-L1 inhibitor, durvalumab, in combination with a PARP inhibitor, olaparib, and a VEGFR1–3 inhibitor, cediranib, in recurrent women’s cancers with biomarker analyses. J. Immunother. Cancer.

[128] André T., Shiu K.-K., Kim T.W., Jensen B.V., Jensen L.H., Punt C., Smith D., Garcia-Carbonero R., Benavides M., Gibbs P. (2020). Pembrolizumab in Microsatellite-Instability–High Advanced Colorectal Cancer. N. Engl. J. Med..

[129] Le D.T., Durham J.N., Smith K.N., Wang H., Bartlett B.R., Aulakh L.K., Lu S., Kemberling H., Wilt C., Luber B.S. (2017). Mismatch repair deficiency predicts response of solid tumors to PD-1 blockade. Science.

[130] Chalabi M., Fanchi L.F., Dijkstra K.K., Van Den Berg J.G., Aalbers A.G., Sikorska K., Lopez-Yurda M., Grootscholten C., Beets G.L., Snaebjornsson P. (2020). Neoadjuvant immunotherapy leads to pathological responses in MMR-proficient and MMR-deficient early-stage colon cancers. Nat. Med..

[131] Mur P., García-Mulero S., del Valle J., Magraner-Pardo L., Vidal A., Pineda M., Cinnirella G., Martín-Ramos E., Pons T., López-Doriga A. (2020). Role of POLE and POLD1 in familial cancer. Genet. Med..

[132] Calegari A.M., Inno A., Monterisi S., Orlandi A., Santini D., Basso M., Cassano A., Martini M., Cenci T., De Pascalis I. (2017). A phase 2 study of temozolomide in pretreated metastatic colorectal cancer with MGMT promoter methylation. Br. J. Cancer.

[133] Wood L.D., Yurgelun M.B., Goggins M.G. (2019). Genetics of Familial and Sporadic Pancreatic Cancer. Gastroenterology.

[134] Armstrong S.A., Schultz C.W., Azimi-Sadjadi A., Brody J.R., Pishvaian M.J. (2019). ATM Dysfunction in Pancreatic Adenocarcinoma and Associated Therapeutic Implications. Mol. Cancer Ther..

[135] Seeber A., Zimmer K., Kocher F., Puccini A., Xiu J., Nabhan C., Elliott A., Goldberg R.M., Grothey A., Shields A.F. (2020). Molecular characteristics of BRCA1/2 and PALB2 mutations in pancreatic ductal adenocarcinoma. ESMO Open.

[136] Waddell N., Pajic M., Patch A.-M., Chang D.K., Kassahn K.S., Bailey P., Johns A.L., Miller D., Nones K., Quek K. (2015). Whole genomes redefine the mutational landscape of pancreatic cancer. Nature.

[137] Flaherty K.T., Gray R.J., Chen A.P., Li S., McShane L.M., Patton D., Hamilton S.R., Williams P.M., Iafrate A.J., Sklar J. (2020). Molecular Landscape and Actionable Alterations in a Genomically Guided Cancer Clinical Trial: National Cancer Institute Molecular Analysis for Therapy Choice (NCI-MATCH). J. Clin. Oncol..

[138] Yarchoan M., Myzak M.C., Johnson B.A., De Jesus-Acosta A., Le D.T., Jaffee E., Azad N.S., Donehower R.C., Zheng L., Oberstein P.E. (2017). Olaparib in combination with irinotecan, cisplatin, and mitomycin C in patients with advanced pancreatic cancer. Oncotarget.

[139] Lowery M.A., Kelsen D.P., Capanu M., Smith S.C., Lee J.W., Stadler Z.K., Moore M.J., Kindler H.L., Golan T., Segal A. (2017). Phase II trial of veliparib in patients with previously treated BRCA-mutated pancreas ductal adenocarcinoma. Eur. J. Cancer.

[140] Pishvaian M.J., Hwang J.J., He A.R., Smaglo B.G., Kim S.S., Weinberg B.A., Weiner L.M., Marshall J.L., Brody J.R. (2020). A Phase I/II Study of Veliparib (ABT-888) in Combination with 5-Fluorouracil and Oxaliplatin in Patients with Metastatic Pancreatic Cancer. Clin. Cancer Res..

[141] Takeuchi S., Doi M., Ikari N., Yamamoto M., Furukawa T. (2018). Mutations in BRCA1, BRCA2, and PALB2, and a panel of 50 cancer-associated genes in pancreatic ductal adenocarcinoma. Sci. Rep..

[142] Golan T., Hammel P., Reni M., Van Cutsem E., Macarulla T., Hall M.J., Park J.-O., Hochhauser D., Arnold D., Oh D.-Y. (2019). Maintenance Olaparib for Germline BRCA-Mutated Metastatic Pancreatic Cancer. N. Engl. J. Med..

[143] Matulonis U.A., Penson R.T., Domchek S.M., Kaufman B., Shapira-Frommer R., Audeh M.W., Kaye S., Molife L.R., Gelmon K.A., Robertson J.D. (2016). Olaparib monotherapy in patients with advanced relapsed ovarian cancer and a germline BRCA1/2 mutation: A multistudy analysis of response rates and safety. Ann. Oncol..

[144] Markham A., Keam S.J. (2020). Selumetinib: First Approval. Drugs.

[145] Ngoi N., Tan D. (2021). The role of homologous recombination deficiency testing in ovarian cancer and its clinical implications: Do we need it?. ESMO Open.

[146] González-Martín A., Pothuri B., Vergote I., DePont Christensen R., Graybill W., Mirza M.R., McCormick C., Lorusso D., Hoskins P., Freyer G. (2019). Niraparib in Patients with Newly Diagnosed Advanced Ovarian Cancer. N. Engl. J. Med..

[147] Coleman R.L., Fleming G.F., Brady M.F., Swisher E.M., Steffensen K.D., Friedlander M., Okamoto A., Moore K.N., Efrat Ben-Baruch N., Werner T.L. (2019). Veliparib with First-Line Chemotherapy and as Maintenance Therapy in Ovarian Cancer. N. Engl. J. Med..

[148] Saner F.A.M., Herschtal A., Nelson B.H., DeFazio A., Goode E.L., Ramus S.J., Pandey A., Beach J.A., Fereday S., Berchuck A. (2019). Going to extremes: Determinants of extraordinary response and survival in patients with cancer. Nat. Cancer.

[149] Jones P.A., Ohtani H., Chakravarthy A., De Carvalho D.D. (2019). Epigenetic therapy in immune-oncology. Nat. Cancer.

[150] Ibrahim Y.H., García-García C., Serra V., He L., Torres-Lockhart K., Prat A., Anton P., Cozar P., Guzmán M., Grueso J. (2012). PI3K Inhibition Impairs BRCA1/2 Expression and Sensitizes BRCA-Proficient Triple-Negative Breast Cancer to PARP Inhibition. Cancer Discov..

[151] Abbotts R., Topper M.J., Biondi C., Fontaine D., Goswami R., Stojanovic L., Choi E.Y., McLaughlin L., Kogan A.A., Xia L. (2019). DNA methyltransferase inhibitors induce a BRCAness phenotype that sensitizes NSCLC to PARP inhibitor and ionizing radiation. Proc. Natl. Acad. Sci. USA.

[152] Sheng W., LaFleur M., Nguyen T., Chen S., Chakravarthy A., Conway J., Li Y., Chen H., Yang H., Hsu P.-H. (2018). LSD1 Ablation Stimulates Anti-tumor Immunity and Enables Checkpoint Blockade. Cell.

[153] Quigley D., Alumkal J.J., Wyatt A.W., Kothari V., Foye A., Lloyd P., Aggarwal R., Kim W., Lu E., Schwartzman J. (2017). Analysis of Circulating Cell-Free DNA Identifies Multiclonal Heterogeneity of BRCA2 Reversion Mutations Associated with Resistance to PARP Inhibitors. Cancer Discov..

